# Deletion of the serine protease CAP2/*Tmprss4* leads to dysregulated renal water handling upon dietary potassium depletion

**DOI:** 10.1038/s41598-019-55995-x

**Published:** 2019-12-20

**Authors:** Anna Keppner, Darko Maric, Chloé Sergi, Camille Ansermet, Damien De Bellis, Denise V. Kratschmar, Jérémie Canonica, Petra Klusonova, Robert A. Fenton, Alex Odermatt, Gilles Crambert, David Hoogewijs, Edith Hummler

**Affiliations:** 1 Department of Pharmacology and Toxicology, University of Lausanne, Lausanne, Switzerland; 2 Department of Medicine/Physiology, University of Fribourg, Fribourg, Switzerland; 3 Department of Pharmaceutical Sciences, University of Basel, Basel, Switzerland; 4 Department of Biomedicine, Aarhus University, Aarhus, Denmark; 5 INSERM, Paris, France; 6 National Center of Competence in Research Kidney Control of Homeostasis (NCCR Kidney.CH), University of Lausanne, Lausanne, Switzerland; 7 Electron Microscopy Facility, University of Lausanne, Lausanne, Switzerland; 8 Department of Plant Molecular Biology, University of Lausanne, Lausanne, Switzerland; 9 Present Address: Ophthalmic Hospital Jules Gonin, University of Lausanne, Lausanne, Switzerland

**Keywords:** Nephrons, Nephrons

## Abstract

The kidney needs to adapt daily to variable dietary K^+^ contents via various mechanisms including diuretic, acid-base and hormonal changes that are still not fully understood. In this study, we demonstrate that following a K^+^-deficient diet in wildtype mice, the serine protease CAP2/*Tmprss4* is upregulated in connecting tubule and cortical collecting duct and also localizes to the medulla and transitional epithelium of the papilla and minor calyx. Male CAP2/*Tmprss4* knockout mice display altered water handling and urine osmolality, enhanced vasopressin response leading to upregulated adenylate cyclase 6 expression and cAMP overproduction, and subsequently greater aquaporin 2 (AQP2) and Na^+^-K^+^-2Cl^−^ cotransporter 2 (NKCC2) expression following K^+^-deficient diet. Urinary acidification coincides with significantly increased H^+^,K^+^-ATPase type 2 (HKA2) mRNA and protein expression, and decreased calcium and phosphate excretion. This is accompanied by increased glucocorticoid receptor (GR) protein levels and reduced 11β-hydroxysteroid dehydrogenase 2 activity in knockout mice. Strikingly, genetic nephron-specific deletion of GR leads to the mirrored phenotype of CAP2/*Tmprss4* knockouts, including increased water intake and urine output, urinary alkalinisation, downregulation of HKA2, AQP2 and NKCC2. Collectively, our data unveil a novel role of the serine protease CAP2/*Tmprss4* and GR on renal water handling upon dietary K^+^ depletion.

## Introduction

Sodium and potassium are essential ions for intra- and extracellular homeostasis. The dietary electrolyte content impacts our health^1^; commonly high sodium and low potassium diets lead to high blood pressure and diuretic use^2^. Renal adaptation to sodium is well studied^3^, while for potassium it still remains largely unknown^4^. Consequently, most anti-hypertensive drugs target sodium transporters. However, disturbances affecting potassium homeostasis are common adverse effects of diuretic use, especially in elderly patients^5^. Hyponatremia, hypokalemia and acid-base disorders (metabolic acidosis) are often found upon hospital admission following adverse diuretic response^5^. The incidence of hospitalized patients with hypokalemia is high^6^ and requires an improved understanding of the renal adaptation mechanisms that prevail under low potassium intake.

The renal adaptations to dietary K^+^ restriction involve a wide variety of mechanisms implicating modified electrolyte and water handling, acid-base components and hormones. Low K^+^ intake increases fractional K^+^ reabsorption in proximal parts of the nephron and reduces delivery to distal parts. Aldosterone secretion decreases, overall reducing Na^+^ reabsorption via the epithelial Na^+^ channel (ENaC) and downregulating the Na^+^-K^+^-2Cl^−^ cotransporter (NKCC2). NKCC2-mediated urinary Na^+^ wasting is avoided by upregulation of the Na^+^-Cl^−^ cotransporter (NCC)^7^. In principal cells (PCs) of the collecting duct, renal outer medullary K^+^ (ROMK) channel-mediated K^+^ secretion diminishes, while reabsorption dramatically increases in intercalated cells (ICs) through increased expression and abundance of the H^+^, K^+^-ATPase (HKA) type 2^8^ thereby promoting urinary acidification^9^, increased calcium^10^ and phosphate excretion^11^. Water handling is modified, characterized by polydipsia and polyuria, likely through diet-induced reduction of vasopressin (AVP)^12^, a stimulator of aquaporin-2 (AQP2) and NKCC2, and/or enhanced autophagic degradation processes^13^.

Serine proteases actively participate in renal electrolyte handling, and in hypertension^14^. Prostasin (CAP1/Prss8) and plasmin were identified as *in vitro* and *in vivo* activators of ENaC^15–18^. Tissue kallikrein not only acts as regulator of ENaC-mediated sodium homeostasis, but also impairs adaptation to high potassium intake in humans, most likely through abnormal activation of HKA2^19^. In rodents, HKA2 is expressed along the nephron in cortical thick ascending limb of Henle’s loop (cTAL), cortical collecting duct (CCD), and outer medullary collecting duct (OMCD), and its expression drastically increases upon K^+^ restriction in CCD^20^ within both A- and B-type ICs, and to a lesser extent in PCs^21^. Global HKA2-deficient mice are unable to retain K^+^ under dietary K^+^ deprivation due to fecal K^+^ wasting^22^. No obvious urinary phenotype was reported under these conditions, although these mice are unable to compensate fecal K^+^ loss by renal K^+^ retention^8,22^. HKA2-deficient mice exhibit defects in urinary circadian excretion of K^+^ leading to instability of kalemia during the nycthemeral cycle^23^ and in pregnancy-induced renal K^+^ retention^24^.

The serine protease CAP2/*Tmprss4* was previously identified as *in vitro* activator of ENaC^25^. However, unlike prostasin and plasmin, CAP2/*Tmprss4* does not participate in ENaC-mediated sodium handling^26^, and further physiological substrates remain unknown. In this study, we show that 1) CAP2/*Tmprss4* expression is regulated by dietary K^+^ intake in specific kidney tubules, and also locates in the medulla and the transitional epithelium lining the papilla and minor calyx; 2) CAP2/*Tmprss4* is implicated in renal adaptation to K^+^ depletion by regulating HKA2, NKCC2 and AQP2; 3) deletion of CAP2/*Tmprss4* is associated with dysregulated GR-mediated signaling, as exemplified by a mirrored phenotype in kidney-specific GR knockout mice. Our results unveil a regulatory function of CAP2/*Tmprss4* and the GR in renal water balance during K^+^ deprivation. These findings may be clinically relevant in conditions resulting in disturbed water handling, as found in nephrogenic diabetes insipidus, Bartter and Gitelman syndromes or in cases of adverse effects following diuretic use.

## Results

### CAP2/Tmprss4 is regulated by dietary K^+^ intake and determines the expression of HKA2

**Figure 1:**
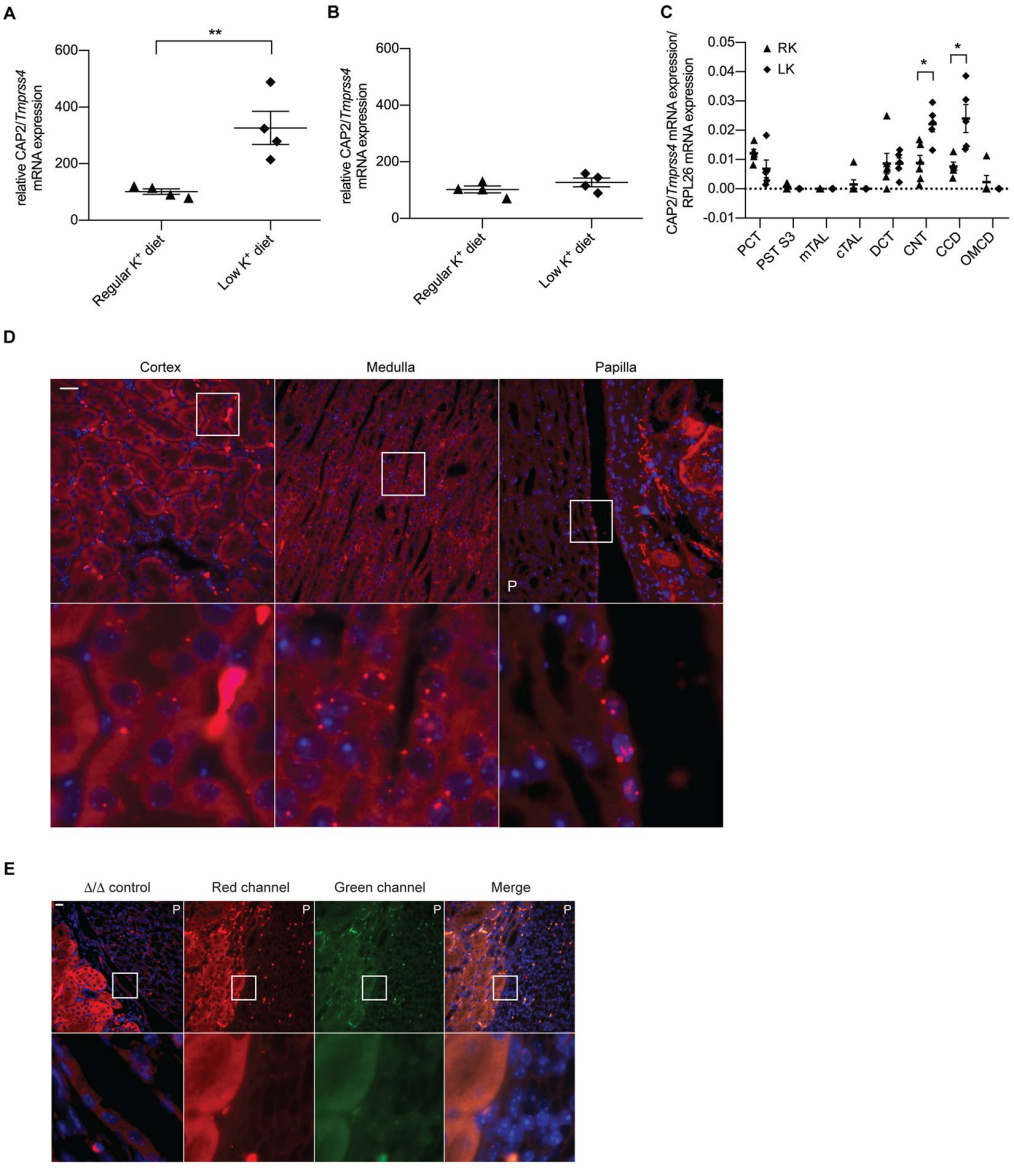
CAP2/*Tmprss4* mRNA expression is upregulated by low dietary K^+^ in distal tubules, and also localizes to the papillary transitional epithelium. Relative mRNA transcript expression levels of CAP2/*Tmprss4* in (**A**) kidney, and (**B**) colon from wildtype mice under regular K^+^ diet (n = 4, triangles) and low K^+^ diet (n = 4, diamonds). (**C**) Detection of wildtype CAP2/*Tmprss4* mRNA transcript expression in microdissected nephron segments (n = 4–6/segment) on regular (RK) and low (LK) potassium diet. PCT: proximal convoluted tubule, PST S3: proximal straight tubule segment 3, mTAL: medullary thick ascending limb of Henle’s loop, cTAL: cortical thick ascending limb of Henle’s loop, DCT: distal convoluted tubule, CNT: connecting tubule, CCD: cortical collecting duct, OMCD: outer medullary collecting duct. (**D**) RNAscope detection of CAP2/*Tmprss4* in renal cortex, medulla and papilla of wildtype mice following LK diet. (**E**) Negative control for CAP2/*Tmprss4* RNAscope detection in knockout (∆/∆) kidney under low K^+^ diet, and negative control for RNAscope fluorescent channels (including channels shown in Fig. 3E,F) in kidney sections from wildtype mice under low K^+^ diet. Magnification 40×, the white boxes indicate zones of higher (63×) magnification, “P” indicates the location of the renal pyramid, scale bar represents 25 µm. * p < 0.05, **p < 0.01.

To assess if CAP2/*Tmprss4* is regulated by dietary K^+^ levels, wildtype male mice were subjected to regular K^+^ diet (RK) or low K^+^ diet (LK). LK diet increased CAP2/*Tmprss4* mRNA expression in kidney but not in colon of wildtype mice (Fig. 1A,B). Renal CAP2/*Tmprss4* mRNA expression was detected in microdissected proximal convoluted tubule (PCT), distal convoluted tubule (DCT), connecting tubule (CNT), and CCD, moderately in proximal straight tubule segment 3 (PST S3) and cTAL, with no detectable signal in medullary thick ascending limb of Henle’s loop (mTAL) and OMCD (Fig. 1C). Following LK diet, expression of CAP2/*Tmprss4* increased significantly in CNT and CCD (Fig. 1C). Cortical expression was confirmed by RNAscope-based CAP2/*Tmprss4* detection, which further revealed additional strong expression in the columnar epithelium of the renal pyramid and papilla, in the transitional epithelium lining the papilla and minor calyx (Fig. 1D) and in single cells along the papillary collecting ducts (Fig. 1D), with no signal in the negative control (Fig. 1E).

**Figure 2:**
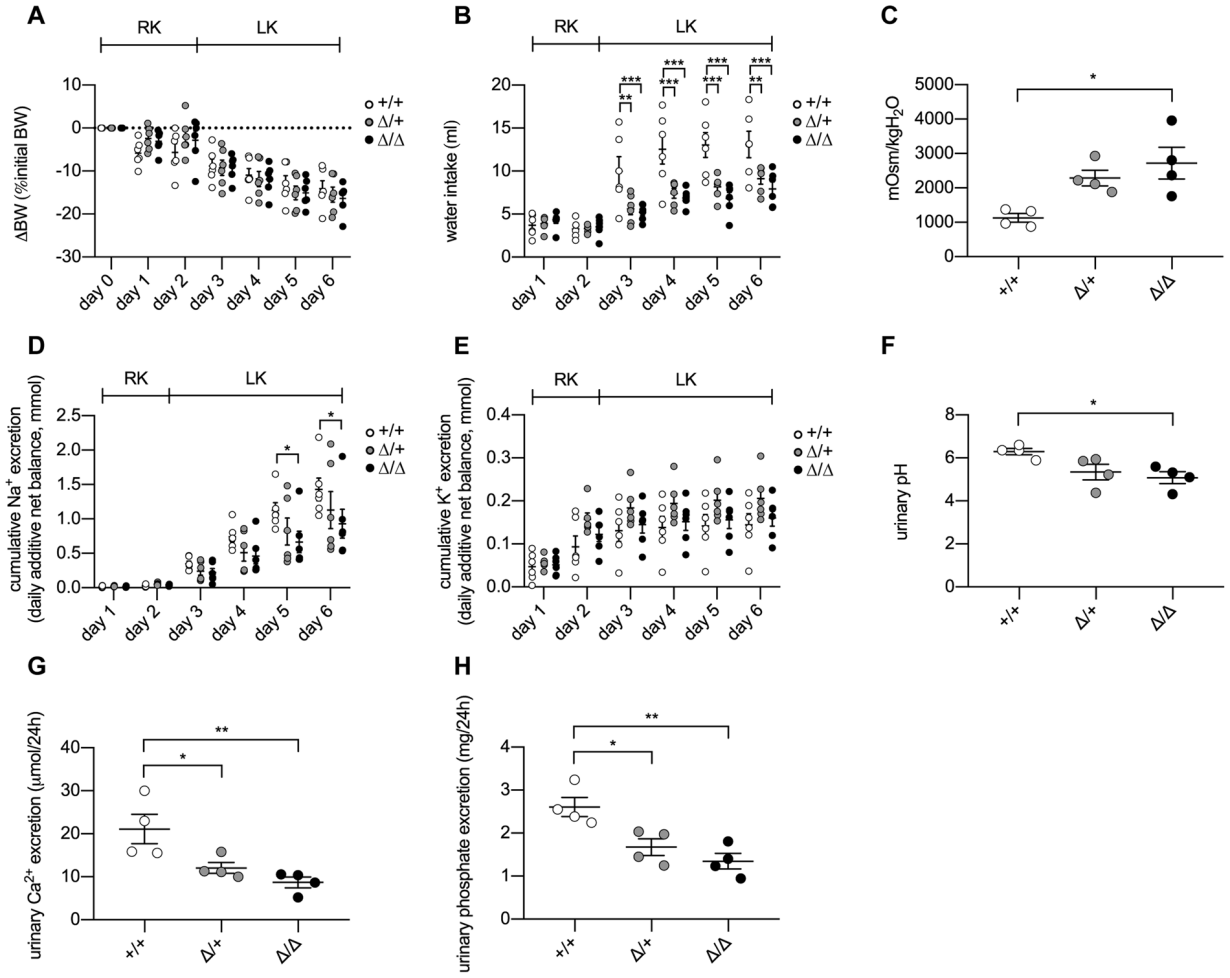
CAP2/*Tmprss4* knockout mice display reduced water intake and urine output on low K^+^ diet. Physiological parameters measured following 2 days under regular K^+^ and 4 days under low K^+^ diet (n = 6 per genotype). (**A**) ∆ body weight (BW) as % of initial BW (g), (**B**) water intake (ml), (**C**) urine osmolality, (**D**) cumulative urinary Na^+^ excretion (mmol), (**E**) cumulative urinary K^+^ excretion (mmol), (**F**) urinary pH, (**G**) 24 h urinary calcium excretion (µmol/24 h), and (**H**) 24 h urinary phosphate excretion (mg/24 h) from CAP2/*Tmprss4* wildtype (+/+, white circles), heterozygous mutant (∆/+, grey circles), and knockout (∆/∆, black circles) mice. *p < 0.05, **p < 0.01, ***p < 0.001.

When feeding CAP2/*Tmprss4* mice with LK diet, all groups similarly lost body weight (Fig. 2A) likely due to the metabolic cage exposure, however both heterozygous and knockout mice equally displayed significantly decreased water intake and urine output (Fig. 2B, Table 1), whereby urine osmolality increased significantly only in knockouts (Fig. 2C). The daily urinary Na^+^ excretions tended to decrease in knockouts, reaching significance on day 5 (Table 1, Fig. S1A, Table S1), leading to a significant reduction of the cumulative urinary Na^+^ excretion from day 5 onwards in knockout mice (Fig. 2D, Table 1). No significant urinary Na^+^ change was observed in heterozygotes (Fig. 2D). The daily urinary K^+^ excretions and the cumulative urinary K^+^ excretion remained statistically unchanged between genotypes (Fig. 2E, Table 1, Fig. S1B, Table S2). The fecal K^+^ excretion, and plasma Na^+^, K^+^, aldosterone concentration and osmolality remained comparable between genotypes (Table 1). Urinary pH was significantly lower in knockout mice (Fig. 2F), together with decreased urinary calcium and phosphate excretion in heterozygous and knockout animals (Fig. 2G,H, Table 1).

**Table 1:** Physiological parameters of CAP2/*Tmprss4* mice after 4 days under low K^+^ diet.

Parameters	K^+^-deficient diet		
	+/+	∆/+	∆/∆
n	6	6	6
Body weight (g)	25.01 ± 3.11	22.32 ± 3.58	20.72 ± 4.46
Food intake (g)	2.44 ± 0.14	2.38 ± 0.10	1.94 ± 0.23
Water intake (ml)	13.10 ± 1.54	9.12 ± 0.62^**^	7.94 ± 0.81^***^
Urine volume (ml)	6.30 ± 0.63	3.69 ± 0.75^**^	2.21 ± 0.51^***^
Urine/water ratio	0.43 ± 0.05	0.41 ± 0.07	0.36 ± 0.08
Feces (g)	0.33 ± 0.03	0.35 ± 0.02	0.27 ± 0.03
Urinary Na^+^ (mmol)	0.30 ± 0.05	0.31 ± 0.08	0.27 ± 0.06
Urinary K^+^ (mmol)	0.03 ± 0.01	0.05 ± 0.01	0.05 ± 0.01
Cumulative Na^+^ (mmol)	1.42 ± 0.16	1.13 ± 0.27	0.93 ± 0.21
Cumulative K^+^ (mmol)	0.14 ± 0.03	0.21 ± 0.02	0.16 ± 0.02
Urinary calcium (µmol/24 h)	20.59 ± 4.42	12.80 ± 1.72	9.44 ± 1.75
Urinary phosphate (mg/24 h)	2.66 ± 0.38	1.77 ± 0.11	1.36 ± 0.32
24 h fecal K^+^ excretion (µmol)	10.21 ± 2.73	12.27 ± 1.98	10.80 ± 2.03
Plasma Na^+^ (mmol/l)	158.03 ± 2.80	150.51 ± 5.52	150.10 ± 1.42
Plasma K^+^ (mmol/l)	3.52 ± 0.20	3.21 ± 0.20	3.82 ± 0.41
Aldosterone (nM)	0.15 ± 0.06	0.24 ± 0.08	0.26 ± 0.06
Plasma osmolality (mOsm/kgH_2_O)	268.17 ± 8.85	261.02 ± 7.68	257.20 ± 2.27

**Figure 3:**
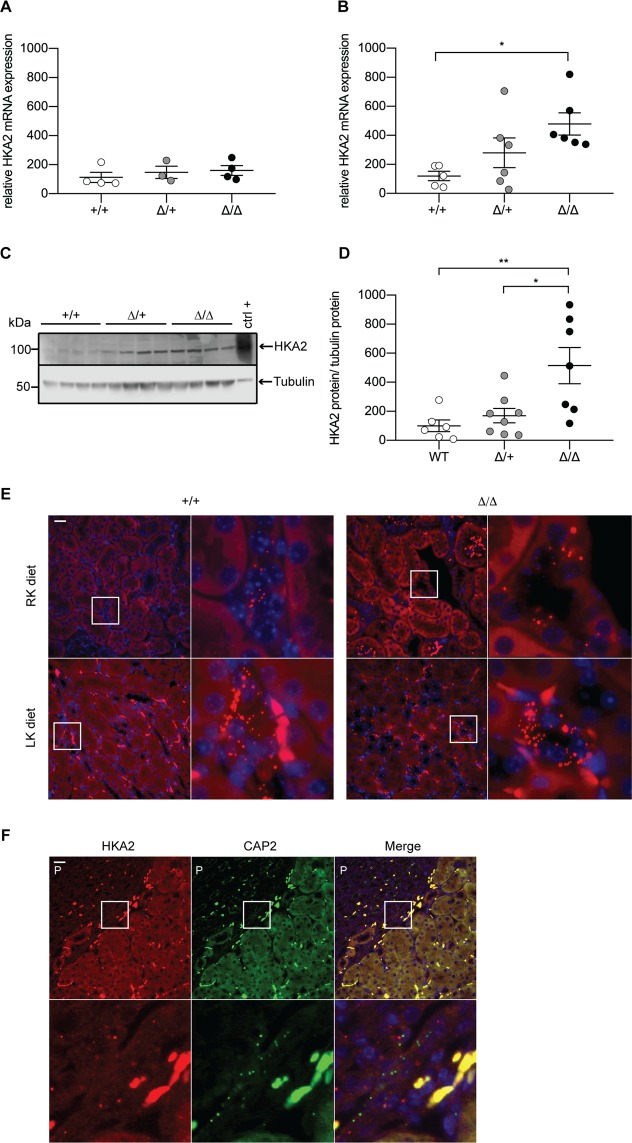
H^+^,K^+^-ATPase type 2 expression is increased in CAP2/*Tmprss4* mice on low K^+^ diet. Relative mRNA transcript expression of H^+^,K^+^-ATPase type 2 (HKA2) in CAP2/*Tmprss4* wildtype (+/+, white circles), heterozygous mutant (∆/+, grey circles), and knockout (∆/∆, black circles) kidneys under (**A**) regular K^+^ diet (n = 5 per genotype), and (**B**) under low K^+^ diet (n = 6 per genotype). (**C**) Representative (cropped) immunoblot for HKA2 in kidney lysates from CAP2/*Tmprss4* wildtype (+/+), heterozygous mutant (∆/+), and knockout (∆/∆) mice under low K^+^ diet (n = 6 per genotype), and (**D**) corresponding protein quantification. A wildtype colon lysate under regular K^+^ diet was used as positive control (ctrl+), and tubulin was used as loading control. The membrane was cut and HKA2 and tubulin were blotted separately. (**E**) RNAscope detection of HKA2 in renal cortex of wildtype and knockout mice under regular K^+^ diet (RK diet, upper panels) and low K^+^ diets (LK diet, lower panels). (**F**) RNAscope co-detection of HKA2 (left panels, red) and CAP2/*Tmprss4* (middle panels, green) and merged pictures (right panels) at the cortico-papillary junction under low K^+^ diet. Magnification 40×, the white boxes indicate zones of higher (63×) magnification, “P” indicates the location of the renal pyramid, scale bar represents 25 µm. *p < 0.05, **p < 0.01. Full-length immunoblots can be found in the supplementary information.

To determine whether urinary acidification and efficient K^+^ retention in CAP2/*Tmprss4* knockout mice under LK diet are linked to altered renal H^+^ and K^+^ channel or transporter expression, we measured the mRNA levels of HKA1, ROMK1 and ROMK2, Big K^+^ channels 1 and 4 (BKβ1 and BKβ4), and two subunits of the vacuolar H^+^-ATPase (ATP6V0A4 and ATP6V1B1). Our results show no significant differences between genotypes (Fig. S2A–G). We next analyzed renal HKA2 mRNA levels. Under RK diet, no difference was detected (Fig. 3A), whereas the mRNA and protein levels of HKA2 were significantly increased in CAP2/*Tmprss4* knockout mice under LK diet (Fig. 3B–D). We confirmed diet-induced expression of HKA2 mRNA in CCD (Fig. 3E), and a similar expression pattern of CAP2/*Tmprss4* at the cortico-pyramidal junction area (Fig. 3F).

In summary, CAP2/*Tmprss4* is regulated by dietary potassium intake, and its deletion in mice subjected to LK diet results in reduced urinary pH possibly linked to increased HKA2 expression.

### Absence of CAP2/Tmprss4 enhances urine osmolality due to maximal renal vasopressin response

**Figure 4:**
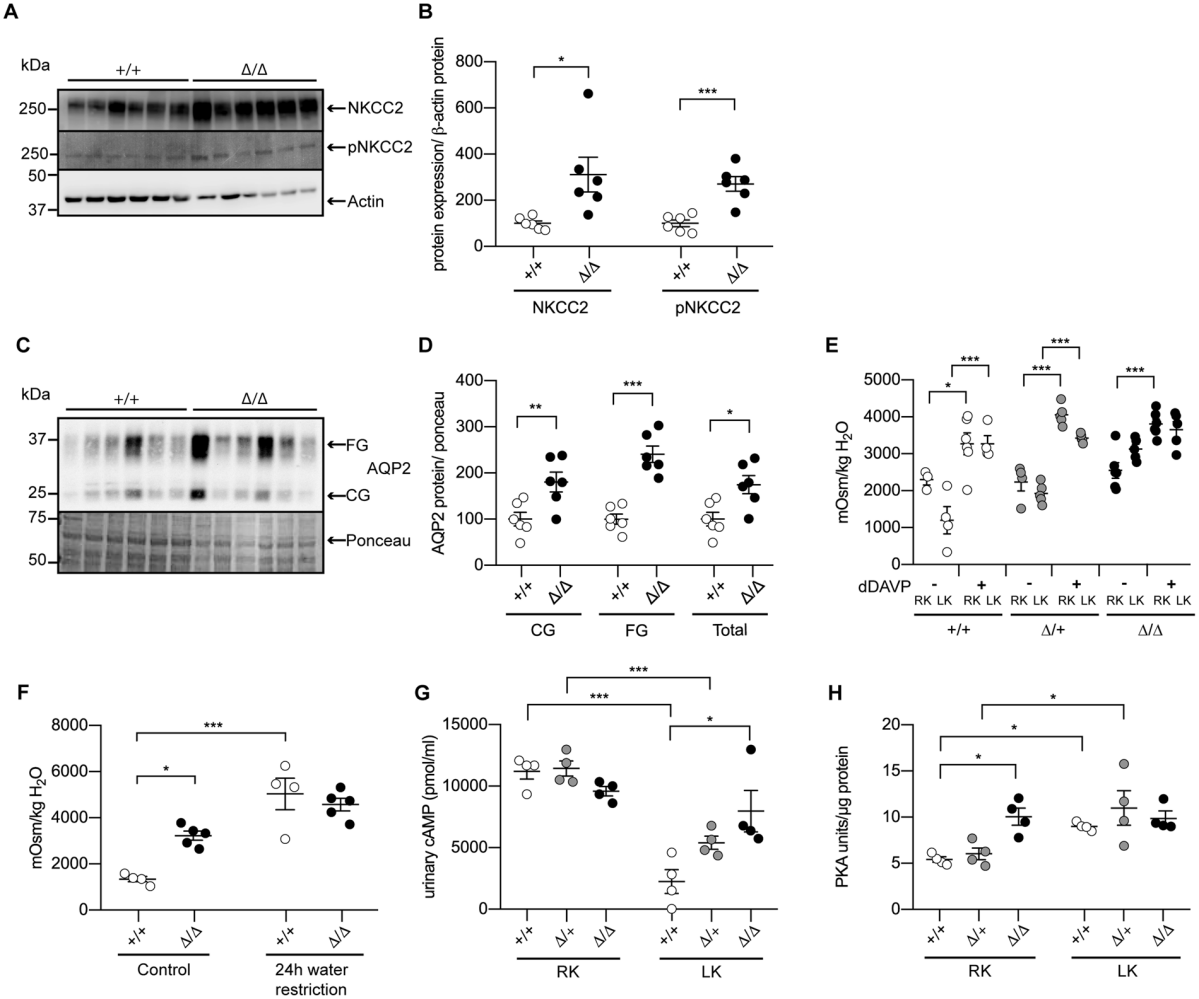
CAP2/*Tmprss4* mice exhibit increased NKCC2 and AQP2 expression and maximal response to dDAVP on low K^+^ diet independently of vasopressin concentration. (**A**) Representative (cropped) immunoblot for NKCC2 and pT96/T101-NKCC2 in kidney lysates from CAP2/*Tmprss4* wildtype (+/+) and knockout (∆/∆) mice under low K^+^ diet (n = 6 per genotype), and (**B**) corresponding protein quantification. (**C**) Representative (cropped) immunoblot for core (CG) and fully glycosylated (FG), and total (CG + FG) aquaporin 2 (AQP2) and (**D**) their corresponding protein quantification in kidney lysates from CAP2/*Tmprss4* wildtype (+/+) and knockout (∆/∆) mice under low K^+^ diet (n = 6 per genotype). Actin was used as loading control for NKCC2 and pNKCC2. The membrane was cut, the lower part was blotted against actin, while the upper part was first blotted against pNKCC2 then stripped and then blotted against NKCC2. The ponceau staining was used as loading control for AQP2. (**E**) Urine osmolality measured before and 5 h after injection of dDAVP in CAP2/*Tmprss4* wildtype (+/+, white circles), heterozygous (∆/+, grey circles) and knockout (∆/∆, black circles) under regular K^+^ diet (RK) and low K^+^ diet (LK) (n = 6 per genotype and per condition). (**F**) Urine osmolality before (control) and after 24 h water restriction in CAP2/*Tmprss4* wildtype (+/+) and knockout (∆/∆) mice under low K^+^ diet (n = 6 per genotype and per condition). (**G**) Urinary cAMP levels in CAP2/*Tmprss4* wildtype (+/+, white circles), heterozygous mutant (∆/+, grey circles), and knockout (∆/∆, black circles) under regular K^+^ diet (RK) and under low K^+^ diet (LK), and (**H**) tissue PKA levels of the corresponding mice. *p < 0.05, **p < 0.01, ***p < 0.001. Full-length immunoblots can be found in the supplementary information.

We next assessed a potential molecular basis for the increased water intake and reduced Na^+^ excretion and detected a significant increase for NKCC2 (mRNA and protein), its phosphorylated form (pT96/101-NKCC2)^27^ (Figs. 4A,B and S3A), and for AQP2 (Fig. 4C,D). AQP3 and AQP4 mRNA levels were increased and reduced, respectively, in CAP2/*Tmprss4* knockout mice (Fig. S3B). We furthermore could detect a significant increase in the mRNA expression of adenylate cyclase 6 (AC6), while the levels of AC5 and the soluble AC (sAC) were comparable between genotypes (Fig. S3C). The mRNA and protein levels of ENaC subunits *Scnn1a*, *Scnn1b* and *Scnn1g* **(**Fig. S4A–E**)**, and protein levels of Na^+^-H^+^ exchangers 1 and 3 (NHE1 and NHE3) **(**Fig. S4F–H**)**, Na^+^, K^+^-ATPase (NKA), NCC and its phosphorylated form (pT53-NCC) (Fig. S5A–C) as well as WNK4 and GILZ mRNA levels did not differ between the groups (Fig. S5D,E). We next investigated the effect on urine osmolality of injection of dDAVP, a V2 receptor agonist, under RK and LK diets. dDAVP led to increased urine osmolality in all groups under RK diet, and in wildtypes and heterozygotes under LK diet (Fig. 4E). In knockout mice, the urine osmolality was not further increased (Fig. 4E) suggesting that the renal response to AVP is maximal in knockout mice under LK diet. Copeptin protein expression, a stable surrogate of AVP, was however unchanged between genotypes (Fig. S3D), neither were mRNA levels of the vasopressin receptors 1a and 2 (avpr1a and avpr2) (Fig. S3E,F). 24 h water restriction under LK diet significantly increased urine osmolality in wildtypes, but similarly to the response to dDAVP treatment, had no effect in knockouts (Fig. 4F). Since AVP-mediated signaling partially depends on cAMP and PKA, indeed wildtype and heterozygous mice displayed strongly reduced urinary cAMP levels following LK diet, while knockout levels remained interestingly elevated (Fig. 4G). Inversely, while tissue PKA levels increased after LK intake in wildtypes and heterozygotes, knockout mice already showed maximal levels under RK diet (Fig. 4H).

To summarize, under LK diet, CAP2/*Tmprss4* knockout mice displayed decreased sensitivity to dDAVP, linked to reduced diuresis and increased urinary Na^+^ retention most likely through increased AQP2, NKCC2 and pNKCC2 protein expression, respectively, and possibly as a consequence of dysregulated AC6 and downstream cAMP and PKA signaling.

### The GR is down-regulated by low dietary K^+^ intake, but remains upregulated following deletion of CAP2/Tmprss4

**Figure 5:**
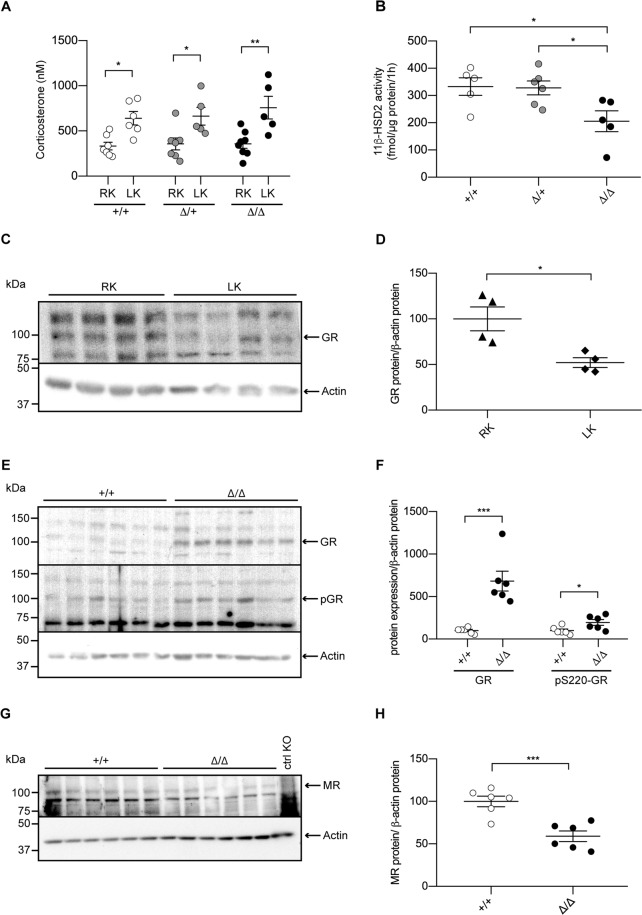
Inadequate 11β-HSD2 activity and glucocorticoid receptor protein expression in CAP2/*Tmprss4* knockout mice under low K^+^ diet. (**A**) Plasma levels of corticosterone (nM) in CAP2/*Tmprss4* wildtype (+/+, white circles, n = 6), heterozygous mutant (∆/+, grey circles, n = 5), and knockout (∆/∆, black circles, n = 5) mice under regular K^+^ (RK) and low K^+^ diet (LK). (**B**) 11β-HSD2 activity (fmol/µg protein/1 h) in CAP2/*Tmprss4* wildtype (+/+, white circles, n = 6), heterozygous mutant (∆/+, grey circles, n = 5), and knockout (∆/∆, black circles, n = 5) mice under low K^+^ diet. (**C**) Representative (cropped) immunoblot for GR in kidney lysates from wildtype mice under regular K^+^ diet (RK) and under low K^+^ diet (LK) (n = 4 per condition) and (**D**) corresponding GR protein quantification (RK, triangles, and LK, diamonds); actin was used as loading control. The membrane was cut and GR and actin were blotted separately. (**E**) Representative (cropped) immunoblot for glucocorticoid receptor (GR) and pS220-GR in kidney lysates from CAP2/*Tmprss4* wildtype (+/+, white circles) and knockout (∆/∆, black circles) mice (n = 6 per genotype) under low K^+^ diet. Actin was used as loading control. The membrane was cut, the lower part was blotted against actin, while the upper part was first blotted against pS220-GR, then stripped and blotted against GR. (**F**) Corresponding GR and pS220-GR protein quantification. (**G**) Representative (cropped) immunoblot of mineralocorticoid receptor (MR) in kidney lysates from CAP2/*Tmprss4* wildtype (+/+) and knockout (∆/∆) mice (n = 6 per genotype) under low K^+^ diet, and (**H**) corresponding MR protein quantification; actin was used as loading control. The membrane was cut and MR and actin were blotted separately. A nephron-specific MR knockout^79^ kidney lysate was used as negative control (ctrl KO) *p < 0.05, **p < 0.01, ***p < 0.001. For the sake of visual clarity, blots for GR and pS220-GR are shown with a higher contrast, quantifications were however performed on the original blots. Full-length original immunoblots can be found in the supplementary information.

Since low potassium intake elicits hormone-dependent regulatory pathways, we analyzed plasma hormone levels in all groups. The levels of progesterone (Fig. S6A), testosterone (Fig. S6B), androstenedione (Fig. S6C), corticosterone (Fig. 5A), 11-dehydrocorticosterone (Fig. S6D), and 11-deoxycorticosterone (Fig. S6E) were not different between genotypes. However, 11-dehydrocorticosterone and corticosterone tended to increase in CAP2/*Tmprss4* knockout mice compared to wildtype littermates and the ratio between corticosterone and 11-dehydrocorticosterone was significantly increased, indicating decreased 11β-hydroxysteroid dehydrogenase type 2 (11β-HSD2) activity (not shown). Consistently, 11β-HSD2 activity was significantly reduced in knockouts, despite unchanged mRNA levels among genotypes (Figs. 5B and S6F). Strikingly, although significantly downregulated in wildtype mice on LK diet compared to RK diet (Fig. 5C,D), the protein levels of the GR and its phosphorylated form (pS220-GR) remained increased in knockout mice (Fig. 5E,F). Moreover, the protein level of the mineralocorticoid receptor (MR) was significantly decreased in knockout mice (Fig. 5G,H), whereas levels of the progesterone receptor (PRα and PRβ), the androgen receptor (AR) and the different isoforms of the estrogen receptor alpha (ERα) in kidney from CAP2/*Tmprss4* mice under LK diet were unchanged (Fig. S7A–D**)**.

Overall, CAP2/*Tmprss4* deletion is associated with a shift to enhanced GR action, with increased GR protein expression and phosphorylation, decreased 11β-HSD2 activity, and reduced MR expression in the kidney under LK diet.

### Nephron-specific deletion of the GR leads to inadequate water handling, thereby mirroring the phenotype of CAP2/Tmprss4 mice

**Figure 6:**
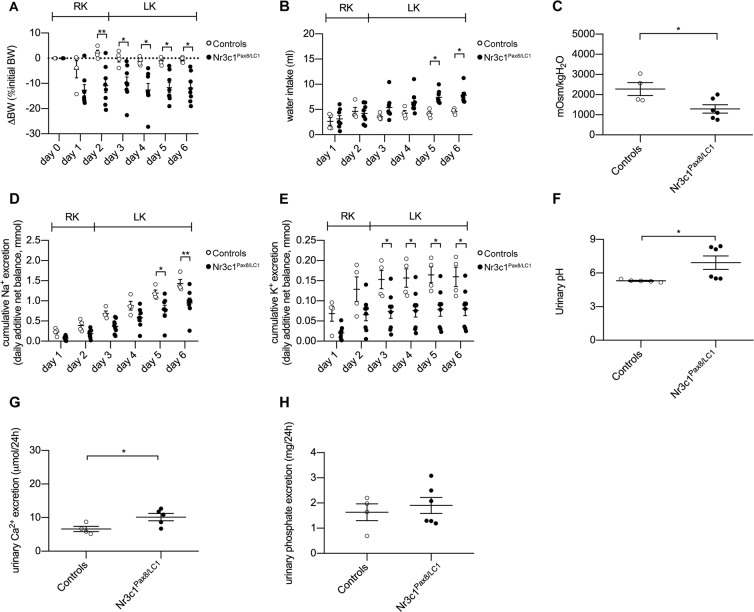
Nephron-specific genetic deletion of GR leads to the mirrored phenotype of CAP2/*Tmprss4* mice with disturbed water, electrolyte and acid-base handling following low K^+^ diet. Physiological parameters measured following 2 days under regular K^+^ and 4 days under low K^+^ diet (n = 4–6 per genotype). (**A**) ∆ body weight (BW) as % of initial BW (g), (**B**) water intake (ml), (**C**) urine osmolality, (**D**) cumulative 24 h urinary Na^+^ excretion (mmol), (E) cumulative 24 h urinary K^+^ excretion (mmol), (**F**) urinary pH, (G) 24 h urinary calcium excretion (µmol/24 h), and (H) 24 h urinary phosphate excretion (mg/24 h) from control (white circles) and Nr3c1^Pax8/LC1^ (black circles) mice. *p < 0.05, **p < 0.01.

**Figure 7:**
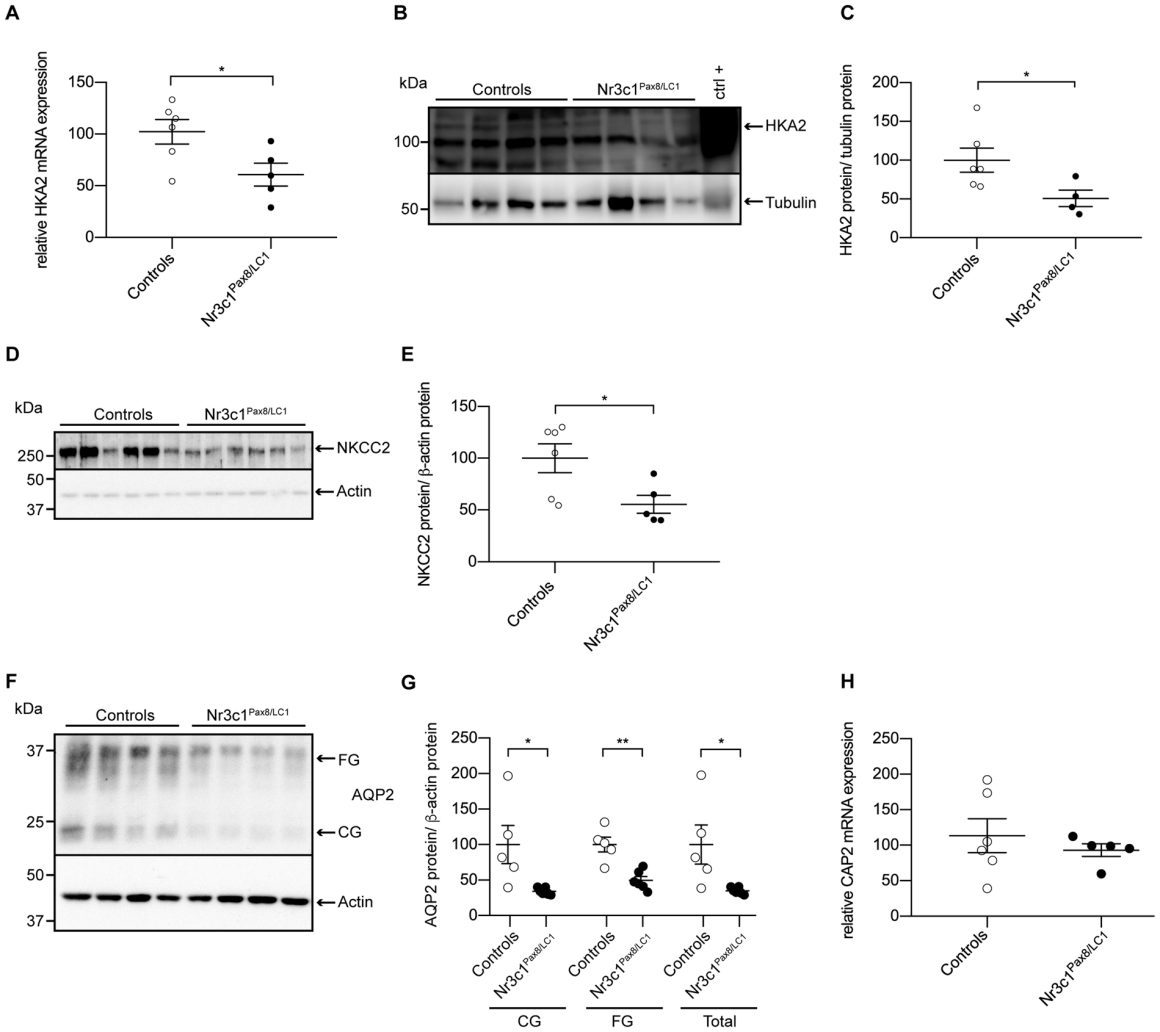
Nephron-specific GR knockout mice display reduced HKA2, NKCC2 and AQP2 protein levels. (**A**) Relative mRNA transcript expression, (**B**) representative (cropped) immunoblot and (**C**) corresponding protein quantification of H^+^,K^+^-ATPase type 2 (HKA2) in control (white circles) and Nr3c1^Pax8/LC1^ (black circles) kidney lysates following low K^+^ diet (n = 4–6 per genotype). A wildtype colon lysate under regular K^+^ diet was used as positive control (ctrl+), and tubulin was used as loading control. The membrane was cut and HKA2 and tubulin were blotted separately. (**D**) Representative (cropped) immunoblot of NKCC2 in control and Nr3c1^Pax8/LC1^ kidney lysates following low K^+^ diet (n = 6 per genotype), and (**E**) corresponding protein quantification. Actin was used as loading control. The membrane was cut and NKCC2 and actin were blotted separately. (**F**) Representative (cropped) immunoblot and (**G**) corresponding protein quantification of AQP2 in control and Nr3c1^Pax8/LC1^ kidney lysates following low K^+^ diet (n = 4–6 per genotype). Actin was used as loading control. The membrane was first blotted against AQP2, then stripped and blotted against actin. (**H**) Relative mRNA transcript expression of CAP2/*Tmprss4* in kidney of control and Nr3c1^Pax8/LC1^ mice following low K^+^ diet (n = 5–6 per genotype). *p < 0.05, **p < 0.01. For the sake of visual clarity, the blot for HKA2 is shown with a higher contrast, quantification was however performed on the original blot. Full-length original immunoblots can be found in the supplementary information.

To study the renal function of the GR upon potassium depletion, we fed inducible nephron-specific GR knockout mice (Nr3c1^Pas8/LC1^)^28^ a LK diet. Knockout animals lost bodyweight, and displayed opposite features to CAP2/*Tmprss4* knockouts, namely increased water intake, increased urine output, reduced urine osmolality, decreased cumulative urinary sodium and potassium excretion, and increased urinary pH and calcium excretion, whereas phosphate excretion was unchanged compared to controls and daily urinary Na^+^ and K^+^ excretions tended to decrease in knockouts (Fig. 6A–H, Table 2, Fig. S1C,D, Tables S3 ans S4). HKA2 mRNA and protein levels, and NKCC2 and AQP2 protein expressions were strongly decreased in Nr3c1^Pax8/LC1^ mice (Fig. 7A–G). CAP2/*Tmprss4* mRNA expression was comparable between genotypes (Fig. 7H).

**Table 2:** Physiological parameters of Nr3c1^Pax8/LC1^ mice after 4 days under low K^+^ diet. “nd” indicates not determined.

Parameters	K^+^-deficient diet	
	Controls	Nr3c1^Pax8/LC1^
n	6	6
Body weight (g)	22.90 ± 0.50	23.49 ± 0.78
Food intake (g)	3.19 ± 0.12	2.98 ± 0.18
Water intake (ml)	4.15 ± 0.36	7.84 ± 0.53^*^
Urine volume (ml)	1.98 ± 0.35	3.88 ± 0.30^*^
Urine/water ratio (ml)	0.41 ± 0.04	0.32 ± 0.05
Feces (g)	0.37 ± 0.04	0.35 ± 0.03
Urinary Na^+^ (mmol)	0.26 ± 0.03	0.17 ± 0.02
Urinary K^+^ (mmol)	0.02 ± 0.01	0.01 ± 0.01
Cumulative Na^+^ (mmol)	1.46 ± 0.13	0.95 ± 0.12^**^
Cumulative K^+^ (mmol)	0.18 ± 0.03	0.08 ± 0.02^*^
Urinary calcium (µmol/24 h)	6.61 ± 0.78	9.64 ± 1.51^*^
Urinary phosphate (mg/24 h)	1.63 ± 0.33	1.90 ± 0.32
Plasma Na^+^ (mmol)	154.2 ± 4.2	156.1 ± 2.2
Plasma K^+^ (mmol)	4.4 ± 0.2	5.9 ± 0.3^***^
Aldosterone (nM)	nd	nd
Plasma osmolality (mOsm/kgH_2_O)	292.67 ± 11.33	309.00 ± 5.29

In summary, our results reveal a novel implication of the serine protease CAP2/*Tmprss4* together with the GR in renal water handling by regulating NKCC2, AQP2 and HKA2 during dietary potassium depletion.

## Discussion

In the present study, we investigated if the serine protease CAP2/*Tmprss4* is implicated in renal adaptation to potassium depletion. Mice deficient for CAP2/*Tmprss4* do not display a visible renal or extrarenal phenotype upon standard and sodium-deficient diets, are healthy and fertile with no urinary phenotype^26^. CAP2/*Tmprss4* mRNA levels were regulated in the kidney, but not in colon of wildtype mice fed with LK diet suggesting a role in renal potassium handling (Fig. 1). Under LK diet, CAP2/*Tmprss4* knockout mice display alterations in their drinking behavior, as characterized by reduced polydipsia and polyuria (Fig. 2 and Table 1) that is normally observed in wildtype animals^29^. Accordingly, following LK diet, the mRNA expression of CAP2/*Tmprss4* increased specifically in the CNT and CCD (Fig. 1), the main sites of fine-tuned water absorption^30^. A recent single-cell RNA sequencing study equally reported strongest expression of CAP2/*Tmprss4* in CCD^31^, however its subcellular localization remains unknown. Intriguingly, RNAscope-based detection also revealed a strong localization of CAP2/*Tmprss4* in the columnar epithelium of the renal pyramid and papilla, in the transitional epithelium lining the minor calyx, and to a lesser extent within the papillary collecting ducts (Fig. 1). The precise function of the renal papilla is still not fully understood, although it is important in urine concentrating mechanisms^32,33^. The pelvocalyceal wall, lining the minor calyx and surrounding the papilla, generates peristaltic contractions, which participate in a mechanical way to fluid reabsorption in the medullary collecting ducts^32^. The absence of CAP2/*Tmprss4* might perturb the solute concentrating mechanisms in the papilla, thereby influencing fluid reabsorption in the papillary collecting ducts where it possibly participates in the fine-tuned control of water balance. As previously described^34^ and observed in CAP2/*Tmprss4* knockout mice (Figs. 2 and 4, Table 1), increased urine osmolality was linked to increased AQP2 and NKCC2 protein expression, thereby enhancing renal water reabsorption and likely being responsible for the observed decreased sodium excretion (Fig. 2, Fig. S1, Table S1). K^+^ depletion normally leads to reduced osmolality and AQP2 and NKCC2 downregulation^35–37^, however, NKCC2, pNKCC2, AQP2 and AQP3 (but not AQP4) were increased in CAP2/*Tmprss4* knockout mice under LK diet (Fig. 4, Fig. S3). Furthermore, sustained dietary K^+^ restriction results in renal AVP insensitivity leading to altered ratio of urine to plasma osmolality^38^. AVP-mediated signaling, controlling both AQP2 and NKCC2^39^, appeared at its maximal capacity in knockouts and accordingly, diet-dependent regulation of cAMP and PKA levels was abrogated (Fig. 4). This may indicate a dysregulation of an adenylate cyclase (AC), thereby altering water homeostasis^40^. Indeed, CAP2/*Tmprss4* knockout mice display a significant increase in the expression of AC6 (Fig. S3), which has been previously described as a major regulator of renal water homeostasis^40^. Interestingly, global knockout mice for AC6 display in contrast to CAP2/*Tmprss4* knockout mice, reduced urine osmolality, lower NKCC2 expression and phosphorylation and strong reductions in the trafficking of AQP2 to the membrane despite increased levels of AVP^41–43^. Furthermore, several studies from the late 70 s and 80 s reported various effects of proteases and their inhibitors on AC activity and subsequent cAMP production^44–47^. Thus, CAP2/*Tmprss4*, via its proteolytic activity, might be a constitutive suppressor of AC6 activity in conditions of strict water regulation such as LK diet, thereby participating in AVP-mediated water homeostasis in distal tubules and in the medulla. Interestingly, publicly available *in silico* tools predict various potential cleavage sites within AC6, including from proteases of the trypsin group of type II transmembrane serine proteases. To summarize, under LK diet, knockout mice displayed maximal AVP-mediated signaling capacity, augmented levels of AC6 leading to increased AQP2 and NKCC2 expression, and likely accounting for the reduced polyuria and increased urine osmolality.

LK diet induced urinary acidification with decreased calcium and phosphate excretions in knockout mice (Fig. 2, Table 1). Simultaneously, knockouts display significantly increased HKA2 mRNA and protein levels (Fig. 3), potentially accounting for the urinary acidification and concurrently supporting K^+^ retention. Interestingly, HKA2 and CAP2/*Tmprss4* localize together along the renal pyramid and papilla (Fig. 3). It is noteworthy that HKA1 can be activated by cAMP^48^, and that similarly the observed rise in HKA2 expression could be a consequence of the augmented cAMP levels (Figs. 3 and 4). Progesterone levels rise during LK diet, enhancing the expression of HKA2 during LK diet^49^ as also observed here (Fig. S6). Steroid hormones were however unchanged (Fig. 5, Fig. S6, Table 1). Interestingly, the activity of the 11β-HSD2 was significantly reduced (Fig. 5). Different studies in human and animals showed increased sodium reabsorption and calcium excretion following inhibition of 11β-HSD2, leading to apparent mineralocorticoid excess syndrome^50^. 11β-HSD2 null mice develop nephrogenic diabetes insipidus (NDI) with sodium wasting, increased diuresis and polydipsia, and reduced urine osmolality and AQP2 expression^51^. 11β-HSD2, AQP2 and NKCC2 dysregulations were further confirmed by transcriptomic studies of NDI mice^52^. The described phenotypes could be secondary to either mineralocorticoid excess, or MR overactivation^50^. However, CAP2/*Tmprss4* knockout mice did not exhibit any of these phenotypes. The highly increased protein levels of the GR and its phosphorylated form S220-GR in knockouts (Fig. 5) can be indicative for GR activation^53^. The observed reduction in MR expression (Fig. 5) might target corticosterone towards GR. Progesterone is also able to antagonize MR activity^54^. Coincidently with the described rise in plasma progesterone levels in wildtype mice under LK diet^49^, increased plasma corticosterone levels were observed in wildtype mice following LK diet (Fig. 5). Several studies reported effects of GR activation on AQP2 and NKCC2 expression, as well as on calcium and phosphate handling^55–57^ It is noteworthy that Elabida and coworkers used the dual GR/PR antagonist RU486 to highlight progesterone actions on HKA2 activity^49^. However, RU486 cross-reacts with GR, thereby further reducing HKA2 expression^49^, as RU486 is also commonly used as GR antagonist in the treatment of Cushing’s syndrome^58,59^, and one side-effect in patients is the reduction of kalemia^58^. However, given the unavailability of a specific PR sparing GR antagonist, we further explored the function of the GR upon K^+^ depletion by using nephron-specific GR knockout mice (Nr3c1^Pax8/LC1^)^28^. Strikingly, GR deletion led to the mirrored phenotype of CAP2/*Tmprss4* knockout mice including HKA2, NKCC2 and AQP2 protein expressions, and additionally impacted body weight and kalemia (Figs. 6, 7, and Table 2). In this context, it is worth mentioning that two potential glucocorticoid response elements are present in the murine HKA2 promoter region^60,61^, and are predicted in the CAP2/*Tmprss4*, NKCC2 and AC6 promoters (UCSC Genome Browser)^61^, although CAP2/*Tmprss4* mRNA expression was unchanged between Nr3c1 control and knockout mice (Fig. 7). Unfortunately, up to date no specific inhibitors are existing and/or commercially available to antagonize specifically either the GR or CAP2/*Tmprss4*. Furthermore, cross-reactivity of progesterone with the GR, or inversely of corticosterone with the PR or the MR, a phenomenon observed in various conditions^62–65^, is not excluded. Indeed, glucocorticoids are described as kaliuretics^66,67^, however the results of the present study, and the observations by Elabida and coworkers^49^, rather suggest a more complex impact of glucocorticoid actions on potassium handling, possibly counter-balanced by PR.

**Figure 8:**
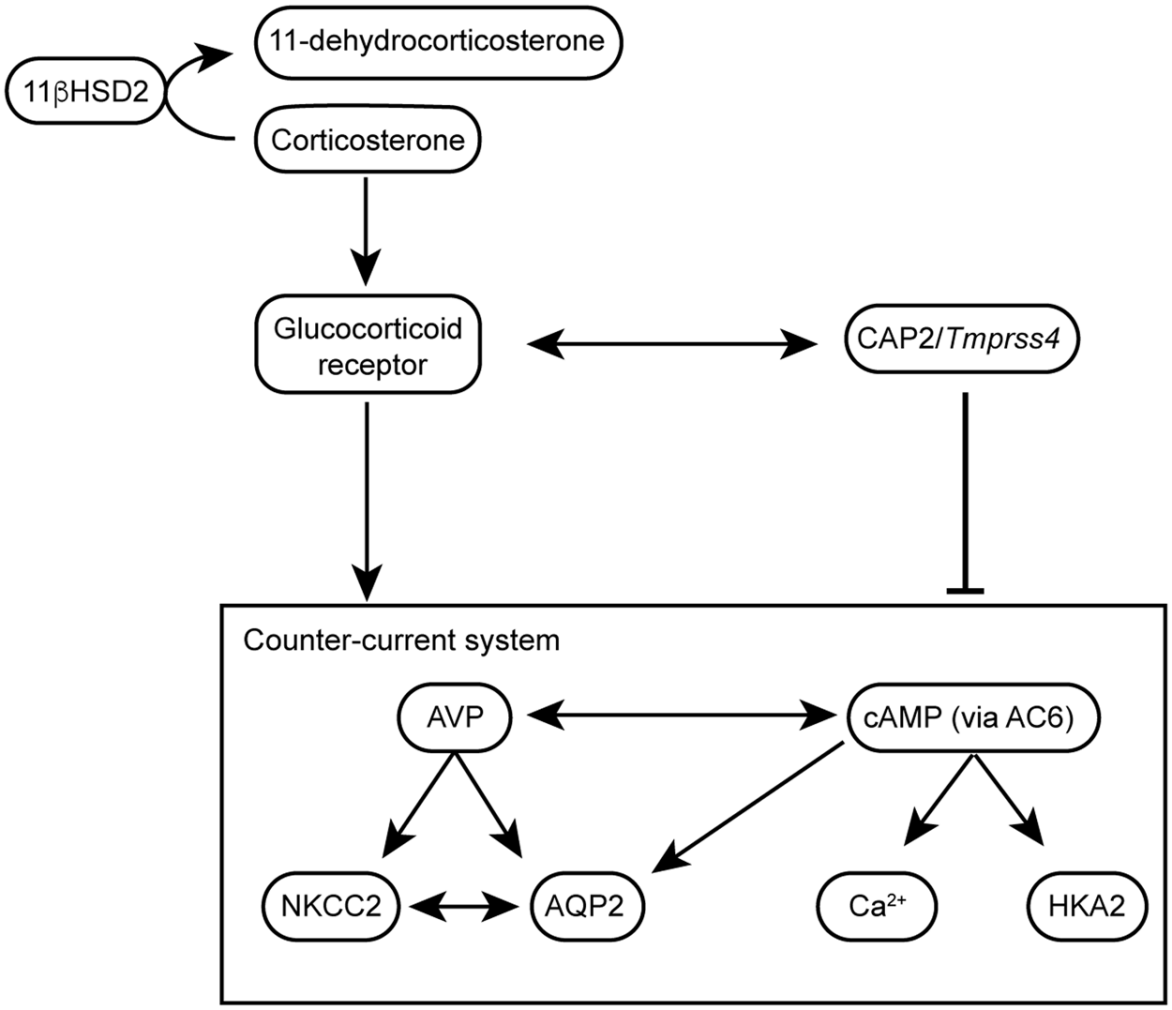
Proposed mechanism of CAP2/*Tmprss4*-mediated water balance in wildtypes during LK diet. During dietary K^+^ depletion in wildtype mice, levels of corticosterone and progesterone normally rise and activity of the 11βHSD2 decreases. In distal tubules, upregulation of CAP2/*Tmprss4* acts negatively on the final expression of NKCC2, AQP2 and HKA2. This may arise through a suppressive function of CAP2/*Tmprss4* on AC6, thereby affecting cAMP-mediated signalling and its downstream effects on NKCC2 and AQP2. The rise is cAMP might also be responsible for increased HKA2 expression, and urinary calcium excretion^38,46^. The GR seems to act positively on these mechanisms, thereby participating in the dysregulated water balance observed in CAP2/*Tmprss4* knockout mice following LK diet.

Taking together the results of our different mouse models, our study shows that the serine protease CAP2/*Tmprss4* is upregulated by LK diet in distal tubules and also locates along the transitional epithelium and in the papilla, where it may proteolytically regulate AC6 activity, thereby affecting water homeostasis by participating in the counter-current regulation (Fig. 8) or the peristaltic contractions modulating the papilla, especially under conditions that require tight control of water reabsorption such as during LK diet. The deletion of CAP2/*Tmprss4* is associated with dysregulated GR-mediated effects (Fig. 8), thereby affecting the normal physiological response to LK diet, further highlighting the crucial endocrine actions for the renal adaptation to LK diet.

In conclusion, this study reveals a novel function for the serine protease CAP2/*Tmprss4* and the GR on renal water handling upon LK intake. CAP2/*Tmprss4* might represent an interesting pharmacological target in diseases modulating diuresis, such as NDI or Bartter’s syndrome, especially in a context of low dietary potassium intake.

## Materials and Methods

### Animals

All experimental procedures and animal maintenance followed Swiss federal guidelines and were approved by the “Direction générale de l’agriculture, de la viticulture et des affaires vétérinaires” of the Canton of Vaud (authorization number VD3333). Animals were housed in rooms with controlled temperature and humidity levels and 12 h/12 h light/dark cycle, with free access to food and water. Age-matched (3–5 months old, C57BL6/N inbred) male CAP2/*Tmprss4* homozygous mutant (CAP2/*Tmprss4*^∆/∆^, ∆/∆, knockout), heterozygous mutant (CAP2/*Tmprss4*^∆/+^, ∆/+) and wildtype (CAP2/*Tmprss4*^+/+^, +/+, WT) littermates^26^ were treated either with regular K^+^ diet (RK, 0.97% K^+^), or low K^+^ diet (LK, <0.003% K^+^) (Ssniff, Spezialdiäten GmbH, Germany). Age-matched (triple transgenic C57BL6/N-mixed background) male GR control mice (Nr3c1^+/+^, controls) and doxycycline-induced kidney-specific knockouts (Nr3c1^Pax8/LC1^) were used^28^. Tubule-specific doxycycline-induced deletion of GR was achieved as previously described^28^. Mice were kept in metabolic cages with free access to food and water for 6 consecutive days, and diet was switched after 2 days from RK to LK. At the end of the experiment, animals were anaesthetized by intraperitoneal injection of a solution containing 10% Rompun (Bayer) and 10% Ketanarkon (Streuli Pharma) diluted in water, blood was collected and animals were sacrificed by cervical dislocation. The 24 h net sodium and potassium excretions (mmol) were calculated by multiplying the concentration (mmol/l) in the collected urine by the urine volume, both collected and assessed during 24 h. Cumulative excretions were calculated by the progressive addition of all the net 24 h excretions for each day over the course of the experiment. Response to dDAVP was performed as previously described^68^.

### Urine and plasma analyses and ELISA

Urine and plasma electrolytes were measured by flame photometry (Instrumentation Laboratory 943 Electrolyte Analyzer, UK). Urinary pH was determined by a pH-meter using fresh urine. Calcium and phosphate were measured at the Zurich Integrative Rodent Physiology facility (ZIRP, University of Zurich, Switzerland) using a UniCel^®^ DxC800 System (Beckman Coulter). Copeptin, cAMP and PKA were measured by ELISA assay (Cloud-Clone Corp., Arbor Assays and Invitrogen respectively).

### Plasma hormone levels and 11β-HSD2 activity

Plasma hormone levels were measured by ultra-pressure LC-MS/MS (UPLC-MS/MS) as described^69^. 11β-HSD2 activity was measured as described^70^.

### RNA extraction and qRT-PCR

Organ preparation, mRNA extraction and cDNA synthesis were performed as described^26^. Real-time PCR was performed using TaqMan Universal PCR Master Mix (Applied Biosystems) or Power SYBRgreen PCR Master Mix (Applied Biosystems), and run using Applied Biosystems 7500 Fast (Carlsbad, CA). Each measurement was performed as duplicate. Quantification of fluorescence was normalized to β-actin. Primer and probe sequences for CAP2/*Tmprss4*, Scnn1a, Scnn1b, Scnn1g, HKA1, HKA2, ROMK1, ROMK2, BKβ1, BKβ4, NKCC2, AQP3, AQP4, GILZ, avpr1a, avpr2, β-actin and gapdh were described^26,51,71–74^, WNK4 transcript expression was determined using TaqMan gene expression assay (Rn00598070).

### Protein extraction, SDS-PAGE and Western blot analysis

Kidneys were homogenized as described^26^. 30 µg of proteins (120 µg for HKA2) were separated by SDS-PAGE on 10% acrylamide gels, and proteins were electrically transferred to nitrocellulose membranes (Amersham Hybond-ECL, GE Healthcare) and incubated overnight at 4 °C with primary antibody against HKA2 (1:200, a gift from Dr. Crambert, INSERM, Paris, France), NKA (1:10000)^75^, NCC (1:500)^76^, pT53-NCC (1:1000)^76^, AQP2 (1:1000, Santa Cruz, Dallas, TX, USA), NKCC2 (1:2000)^77^, pT96/T101-NKCC2 (1:200)^27^, GR (1:1000, Santa Cruz, Dallas, TX, USA), pS211-GR (1:200, Bioss, Boston, MA, USA), PR (1:1000, Santa Cruz, Dallas, TX, USA), AR (1:1000, Santa Cruz, Dallas, TX, USA), ERα (1:200, Santa Cruz, Dallas, TX, USA), MR (1:50)^75^, Scnn1a (1:500)^78^, Scnn1b (1:1000)^78^, Scnn1g (1:1000)^78^, β-actin (1:1000, Sigma-Aldrich) and α tubulin (1:1000, Santa Cruz, Dallas, TX, USA), and for 1 hour with donkey anti-rabbit IgG HRP-conjugated secondary antibody (1:10000, Amersham, Bukinghampshire, UK) or donkey anti-mouse IgG HRP-conjugated (Jackson Immuno Research, Baltimore, PA, USA) (all antibodies in TBS-Tween 1% and dried milk 2%). The signal was revealed using SuperSignal West Dura detection system (Pierce, Rockford, IL) and quantified using ImageStudio^TM^ Lite program (LI-COR).

### RNAscope

RNAscope Multiplex Fluorescent V2 assay (Bio-techne, Ref.323110) was performed according to manufacturer’s protocol on 4 µm paraffin sections, hybridized with probes Mm-Tmprss4-O1 (Bio-techne, Ref.559451), Mm-Atp12a-C2 (Bio-techne, Ref.500571-C2), Mm 2.5 Duplex positive control (Bio-techne, Ref.321651), Duplex negative control DapB (Bio-techne, Ref.320751) at 40 °C for 2 hours and revealed with TSA Opal570 (Perkin Elmer, Ref.FP1488001KT) or TSA Opal520 (Perkin Elmer, Ref.FP1487001KT). Tissues were counterstained with DAPI and mounted with Prolong Diamond Antifade Mountant (Thermo Fisher, P36965). Stainings were performed by the Histology Core Facility of the Ecole Polytechnique Fédérale de Lausanne (EPFL). Negative controls for all channels are shown (Fig. 1E).

### Statistical analysis

Results are presented as mean ± SEM. Data were analyzed by one-way, two-way ANOVA or unpaired two-sample Student’s t-test. P < 0.05 was considered statistically significant.

## Supplementary information

Supplementary information

## Data Availability

All data generated and analyzed in this study are available from the corresponding author upon request.
