# Supplementary material for: Deletion of the serine protease CAP2/Tmprss4 leads to dysregulated renal water handling upon dietary potassium depletion
Source: Sci Rep. 2019 Dec 20;9:19540. doi: 10.1038/s41598-019-55995-x (PMC6925205; doi:10.1038/s41598-019-55995-x)

Deletion of the serine protease CAP2/*Tmprss4* leads to dysregulated renal water handling upon dietary potassium depletion

Anna Keppner[1,2,6], Darko Maric[1,2,6], Chloé Sergi[1], Camille Ansermet[1], Damien De Bellis[1,7,8], Denise V. Kratschmar[3,6], Jérémie Canonica[1,6,9], Petra Klusonova[3,6], Robert A. Fenton[4], Alex Odermatt[3,6], Gilles Crambert[5], David Hoogewijs[2,6], Edith Hummler[1,6]

Supplementary Information

Figure S1. The daily urinary Na^+ and K^+ excretions are only moderately changed in CAP2/*Tmprss4* and nephron-specific GR knockout mice.

Figure S2. mRNA transcript expression of K^+ transporting systems is unchanged in CAP2/*Tmprss4* knockout mice.

Figure S3. NKCC2, AQP3, AQP4 and AC6 mRNA expressions are modified in CAP2/*Tmprss4* knockout mice, independently of AVP and its receptors.

Figure S4. mRNA transcript expression of Na^+ transporting systems is unchanged in CAP2/*Tmprss4* knockout mice.

Figure S5. Na^+,K^+-ATPase and total and phosphorylated NCC are unchanged in CAP2/*Tmprss4* knockout mice.

Figure S6. Plasma hormone levels and 11β-HSD2 mRNA expression in CAP2/*Tmprss4* knockout mice.

Figure S7. Protein expression of steroid hormone receptors in CAP2/*Tmprss4* knockout mice.

Tables S1-S4. Daily urinary Na^+ and K^+ excretions of CAP2/*Tmprss4* and $Nr3c1^{Pax8/LC1}$ mice.

Uncropped Western blots parts 1 to 4

Supplementary figure legends

Figure S1. The daily urinary Na^+ and K^+ excretions are only moderately changed in CAP2/*Tmprss4* and nephron-specific GR knockout mice. Daily urinary Na^+ (**A**) and K^+ (**B**) excretions (mmol/day) in CAP2/*Tmprss4* wildtype (+/+, white circles), heterozygous mutant (Δ/+, grey circles), and knockout (Δ/Δ, black circles) mice (n=6 per genotype) following 2 days under regular K^+ and 4 days under low K^+ diet. Daily urinary Na^+ (**C**) and K^+ (**D**) excretions (mmol/day) in control (white circles) and Nr3c1$^{Pax8/LC1}$ (black circles) mice (n=4-6 per genotype) following 2 days under regular K^+ and 4 days under low K^+ diet. (n=4-6 per genotype). * $p< 0.05$.

Figure S2. mRNA transcript expression of K^+ transporting systems is unchanged in CAP2/*Tmprss4* knockout mice. (**A-E**) Relative mRNA transcript expression levels in kidneys from CAP2/*Tmprss4* wildtype (+/+, white circles), heterozygous mutant (Δ/+, grey circles), and knockout (Δ/Δ, black circles) mice (n=6 per genotype) under low K^+ diet of (**A**) H^+,K^+-ATPase type 1 (HKA1), (**B**) renal outer medullary K^+ channel 1 (ROMK1), (**C**) ROMK2, (**D**) big K^+ channel 1 (BKβ1), (**E**) BKβ4, (**F**) vacuolar H^+-ATPase subunit a4 (ATP6V0A4), and (**G**) subunit b1 (ATP6V1B1).

Figure S3. NKCC2, AQP3, AQP4 and AC6 mRNA expressions are modified in CAP2/*Tmprss4* knockout mice, independently of AVP and its receptors. Relative mRNA transcript expression levels of (**A**) NKCC2, (**B**) AQP3 and AQP4, and (**C**) adenylate cyclase 5 (AC5), 6 (AC6) and the soluble form (sAC) in kidneys from CAP2/*Tmprss4* wildtype (+/+, white circles), heterozygous mutant (Δ/+, grey circles), and knockout (Δ/Δ, black circles) mice (n=4) under low K^+ diet. (**D**) Plasma copeptin levels in CAP2/*Tmprss4* wildtype (+/+, white circles), heterozygous (Δ/+, grey circles) and knockout (Δ/Δ, black circles) under regular K^+

diet (RK) and low K^+ diet (LK) (n=6 per genotype). Relative mRNA expression levels of (**E**) vasopressin receptor 1a (*avpr1a)*, and (**F**) *avpr2* in kidneys from CAP2/*Tmprss4* wildtype (+/+, white circles), heterozygous mutant (Δ/+, grey circles), and knockout (Δ/Δ, black circles) mice (n=4 per genotype) under low K^+ diet. * p<0.05.

Figure S4. mRNA transcript expression of Na^+ transporting systems is unchanged in CAP2/*Tmprss4* knockout mice. Relative mRNA transcript expression levels of (**A**) α-ENaC (Scnn1a), (**B**) β-ENaC (Scnn1b), and (**C**) γ-ENaC (Scnn1g) in kidneys from CAP2/*Tmprss4* wildtype (+/+, white circles), heterozygous mutant (Δ/+, grey circles), and knockout (Δ/Δ, black circles) mice (n=3) under low K^+ diet. (**D**) Representative (cropped) immunoblots for α-ENaC (Scnn1a), β-ENaC (Scnn1b) and γ-ENaC (Scnn1g) in kidney lysates from CAP2/*Tmprss4* wildtype (+/+, white circles), heterozygous mutant (Δ/+, grey circles), and knockout (Δ/Δ, black circles) mice (n=3-4) under low K^+ diet, and (**E**) corresponding protein quantifications for full-length (F) and cleaved (C) subunits. Actin was used as loading control. The membranes were first blotted against the different ENaC subunits, then stripped and blotted against actin. (**F**) Representative immunoblot for Na^+-H^+ exchangers 1 and 3 (NHE1 and NHE3) in kidney lysates from CAP2/*Tmprss4* wildtype (+/+) and knockout (Δ/Δ) mice under low K^+ diet (n=4-6 per genotype). Actin was used as loading control. The membranes were cut, the lower part was blotted against actin, while the upper part was first blotted against NHE1, stripped, and then blotted against NHE3. (**G,H**) Corresponding protein quantification of (**G**) NHE1, and (**H**) NHE3. Full-length blots can be found at the end of this supplementary information file.

Figure S5. Na^+,K^+-ATPase and total and phosphorylated NCC are unchanged in CAP2/*Tmprss4* knockout mice. (**A**) Representative (cropped) immunoblots for Na^+,K^+-ATPase (NKA), NCC, and pT53-NCC in kidney lysates from CAP2/*Tmprss4* wildtype (+/+,

white circles), heterozygous mutant (Δ/+, grey circles), and knockout (Δ/Δ, black circles) mice under low K^+ diet (n=3 per genotype). Actin was used as loading control. For NKA, the membrane was cut, the lower part was blotted against actin, while the upper part was blotted against NKA. For NCC and pT52-NCC, the membrane was cut, the lower part was blotted against actin, while the upper part was first blotted against pT53-NCC, stripped, and then blotted against NHE3. (**B-C**) Corresponding protein quantification of (**B**) NKA and (**C**) NCC and pT53-NCC. (**D-E**) Relative mRNA transcript expression levels of (**D**) WNK lysine deficient protein kinase 4 (WNK4), and (**E**) glucocorticoid-induced leucine zipper (GILZ) in kidneys from CAP2/*Tmprss4* wildtype (+/+, white circles), heterozygous mutant (Δ/+, grey circles), and knockout (Δ/Δ, black circles) mice (n=3-4 per genotype) under low K^+ diet. Full-length blots can be found at the end of this supplementary information file.

Figure S6. Plasma hormone levels and 11β-HSD2 mRNA expression in CAP2/*Tmprss4* knockout mice. Plasma levels (nM) of (**A**) progesterone, (**B**) testosterone, (**C**) androstenedione, (**D**) 11-dehydrocorticosterone, and (**E**) 11-deoxycorticosterone in CAP2/*Tmprss4* wildtype (+/+, white circles, n=6), heterozygous mutant (Δ/+, grey circles, n=5), and knockout (Δ/Δ, black circles, n=5) mice under regular K^+ (RK) and low K^+ diet (LK). (**F**) Relative 11β-HSD2 mRNA expression in CAP2/*Tmprss4* wildtype (+/+, white circles, n=6), heterozygous mutant (Δ/+, grey circles, n=5), and knockout (Δ/Δ, black circles, n=5) kidneys under low K^+ diet. * $p< 0.05$, ** $p< 0.01$.

Figure S7. Protein expression of steroid hormone receptors in CAP2/*Tmprss4* knockout mice. (**A**) Representative (cropped) immunoblots for progesterone receptor α and β (PRα and PRβ), androgen receptor (AR), and the different estrogen receptor α isoforms (ER iso1 55kDa, iso2 51kDa, iso3 46kDa, and iso4 35kDa) in kidney lysates from CAP2/*Tmprss4* wildtype (+/+,

white circles) and knockout (Δ/Δ, black circles) mice (n=4 per genotype) under low K^+ diet; actin was used as loading control. (**B-D**) Corresponding protein quantifications of (**B**) PR, (**C**) AR, and (**D**) ER. *** $p < 0.001$. Full-length blots can be found at the end of this supplementary information file.

Uncropped Western blots. Part 1 contains the full-length original blots for main figures 3C, 4A, 4C, and 5C. Part 2 contains the full-length original blots for main figures 5E, 5G, 7B, 7D, and 7F. Part 3 contains the full-length original blots for supplementary figures S3D, S3F, and S4A. Part 4 contains the full-length original blots for supplementary figure S6A. For each blot, the cropped area is indicated by a black square. Molecular weights are indicated on each blot.

Figure S1

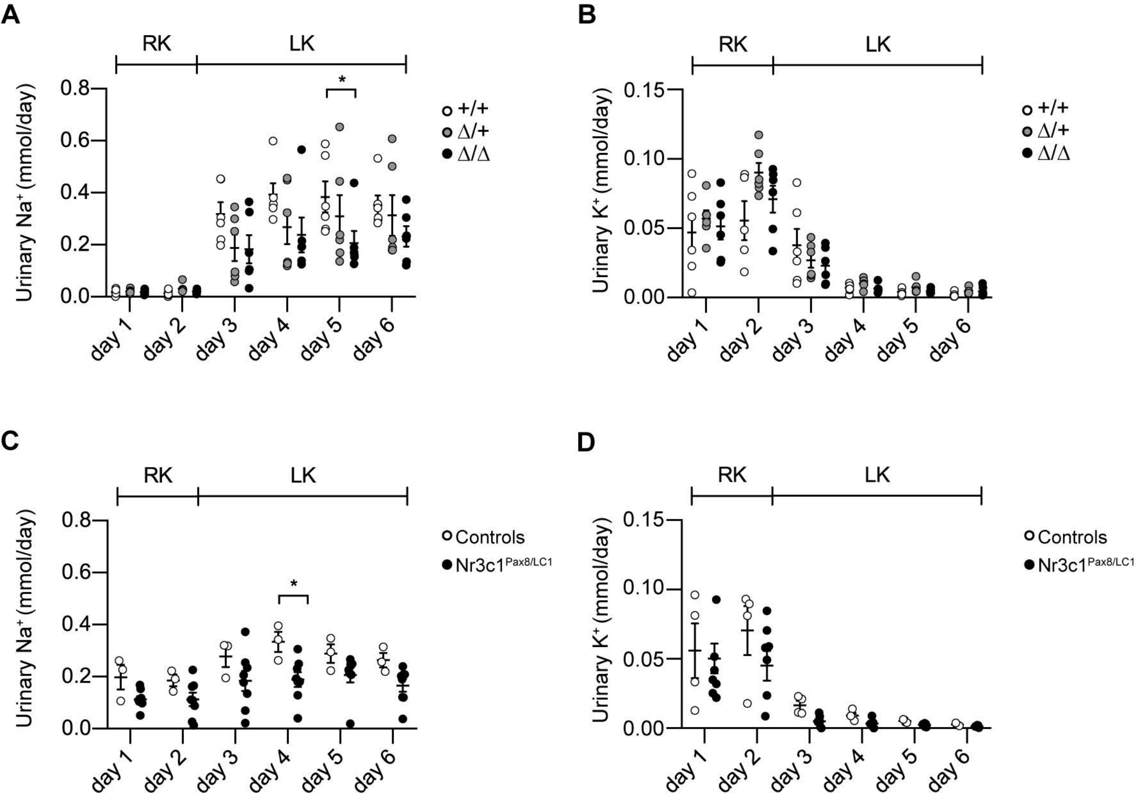

A
RK
LK
*
Urinary Na⁺ (mmol/day)
0.0
0.2
0.4
0.6
0.8
day 1
day 2
day 3
day 4
day 5
day 6
+/+
Δ/+
Δ/Δ
B
RK
LK
Urinary K⁺ (mmol/day)
0.00
0.05
0.10
0.15
day 1
day 2
day 3
day 4
day 5
day 6
+/+
Δ/+
Δ/Δ
C
RK
LK
*
Urinary Na⁺ (mmol/day)
0.0
0.2
0.4
0.6
0.8
day 1
day 2
day 3
day 4
day 5
day 6
Controls
Nr3c1Pax8/LC1
D
RK
LK
Urinary K⁺ (mmol/day)
0.00
0.05
0.10
0.15
day 1
day 2
day 3
day 4
day 5
day 6
Controls
Nr3c1Pax8/LC1

Figure S2

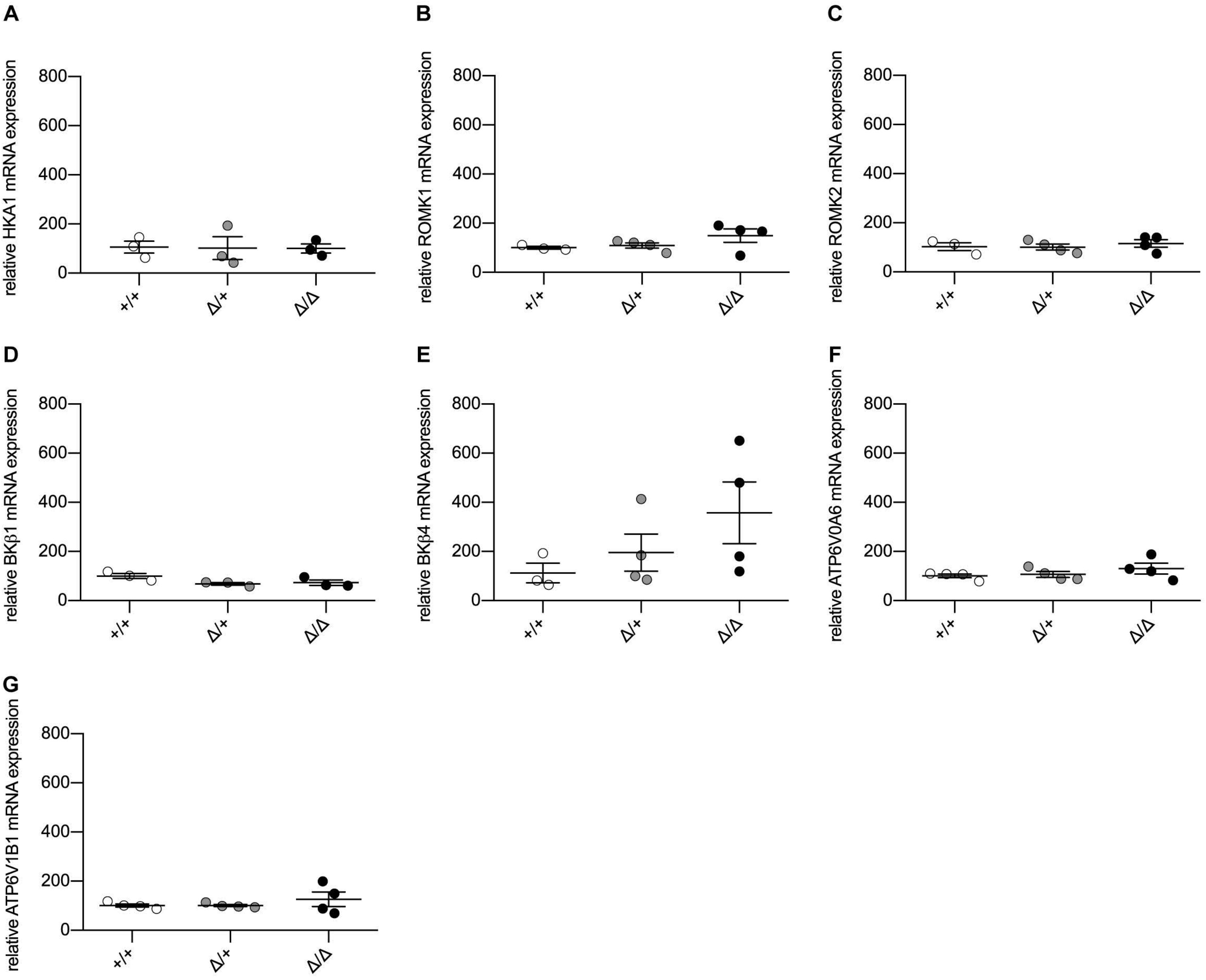

A
B
C
D
E
F
G
relative HKA1 mRNA expression
relative ROMK1 mRNA expression
relative ROMK2 mRNA expression
relative BKβ1 mRNA expression
relative BKβ4 mRNA expression
relative ATP6V0A6 mRNA expression
relative ATP6V1B1 mRNA expression
800
600
400
200
0
+/+
Δ/+
Δ/Δ

Figure S3

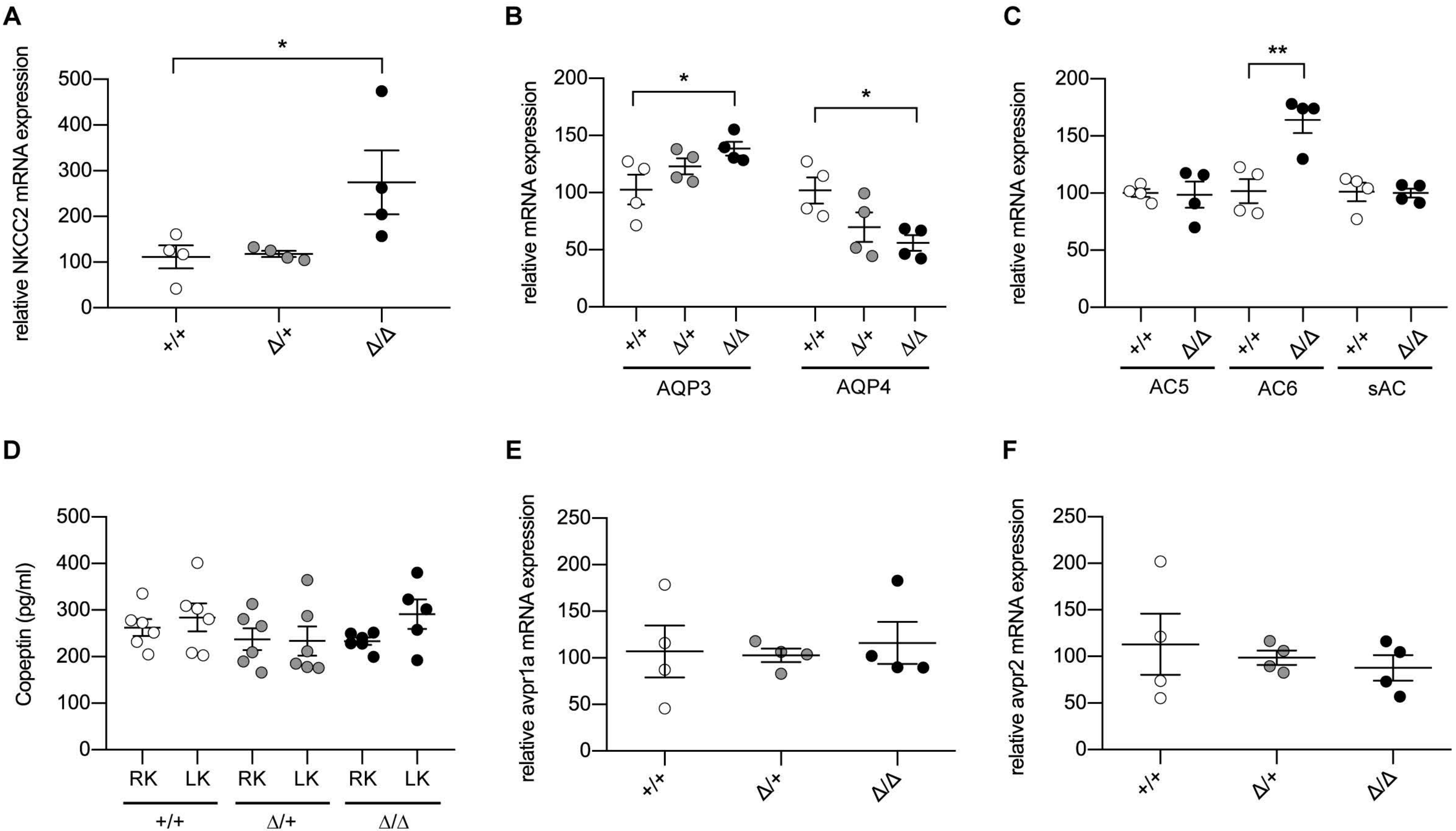
A
B
C
D
E
F
relative NKCC2 mRNA expression
relative mRNA expression
Copeptin (pg/ml)
relative avpr1a mRNA expression
relative avpr2 mRNA expression
+/+
Δ/+
Δ/Δ
AQP3
AQP4
AC5
AC6
sAC
RK
LK
*
**

Figure S4

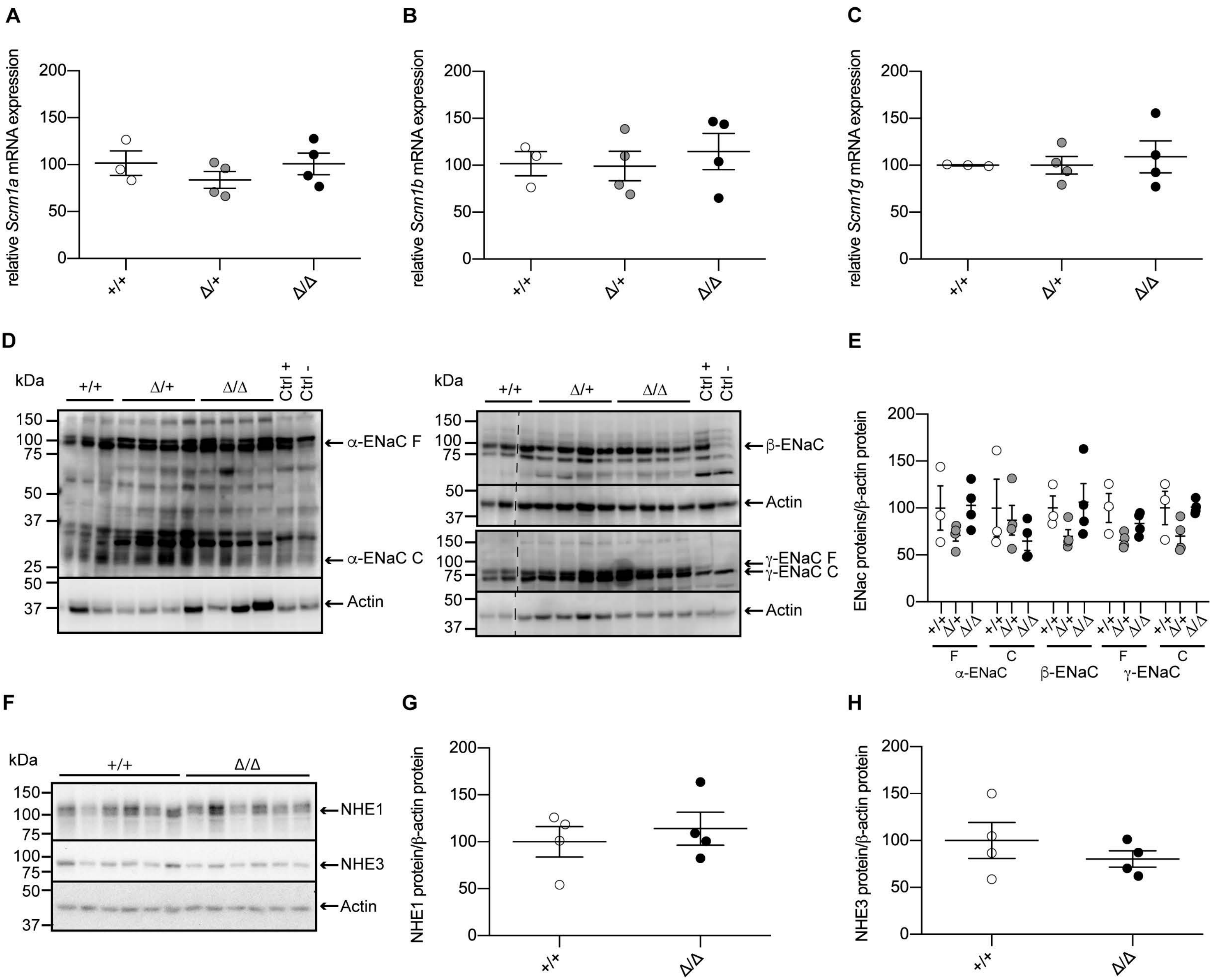

A
relative Scnn1a mRNA expression
+/+
Δ/+
Δ/Δ
B
relative Scnn1b mRNA expression
C
relative Scnn1g mRNA expression
D
kDa
Ctrl +
Ctrl -
α-ENaC F
α-ENaC C
Actin
β-ENaC
γ-ENaC F
γ-ENaC C
E
ENac proteins/β-actin protein
F
C
α-ENaC
β-ENaC
γ-ENaC
F
NHE1
NHE3
G
NHE1 protein/β-actin protein
H
NHE3 protein/β-actin protein

Figure S5

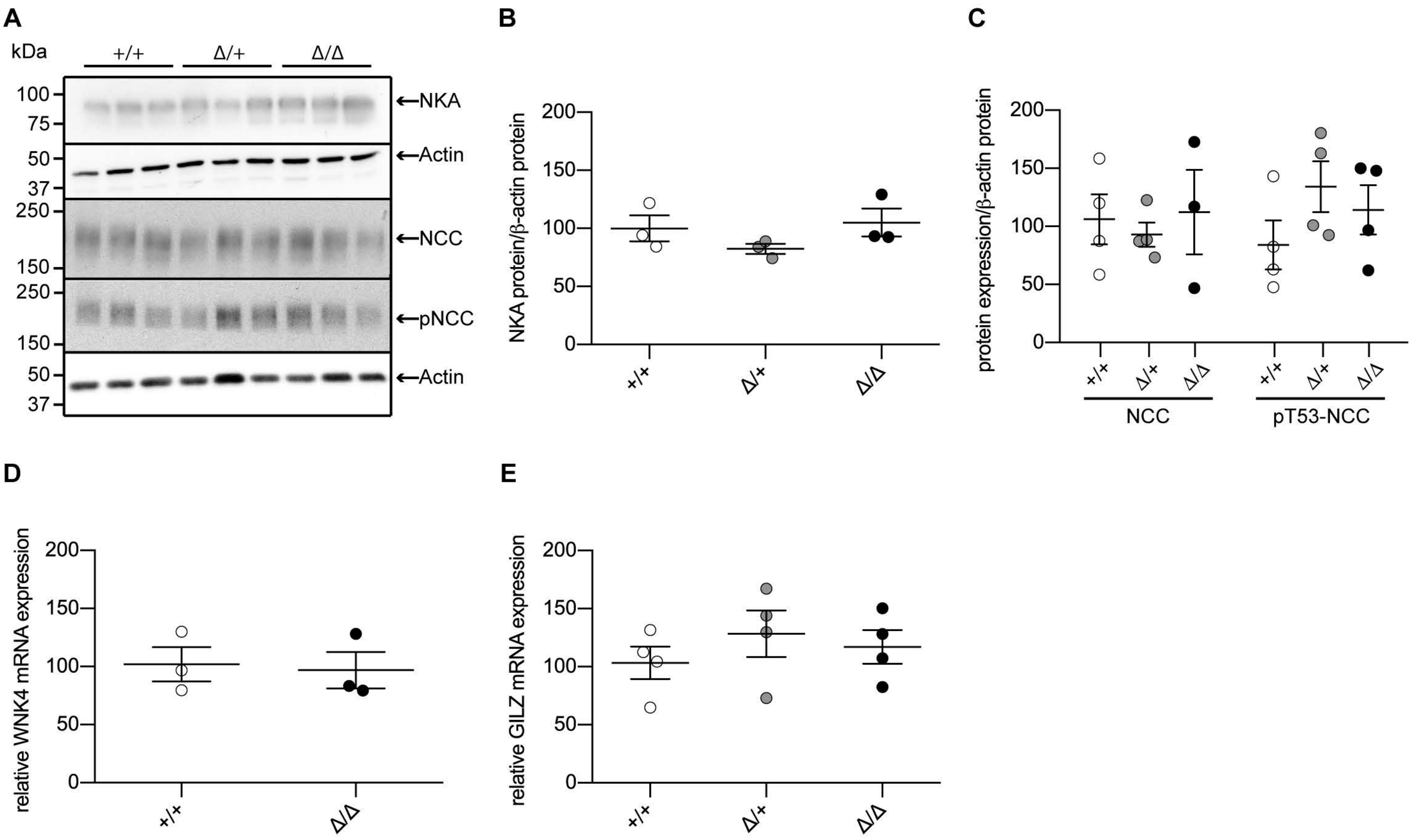
A
kDa
+/+
Δ/+
Δ/Δ
100
75
50
37
250
150
250
150
50
37
←NKA
←Actin
←NCC
←pNCC
←Actin
B
NKA protein/β-actin protein
200
150
100
50
0
+/+
Δ/+
Δ/Δ
C
protein expression/β-actin protein
200
150
100
50
0
+/+
Δ/+
Δ/Δ
+/+
Δ/+
Δ/Δ
NCC
pT53-NCC
D
relative WNK4 mRNA expression
200
150
100
50
0
+/+
Δ/Δ
E
relative GILZ mRNA expression
200
150
100
50
0
+/+
Δ/+
Δ/Δ

Figure S6

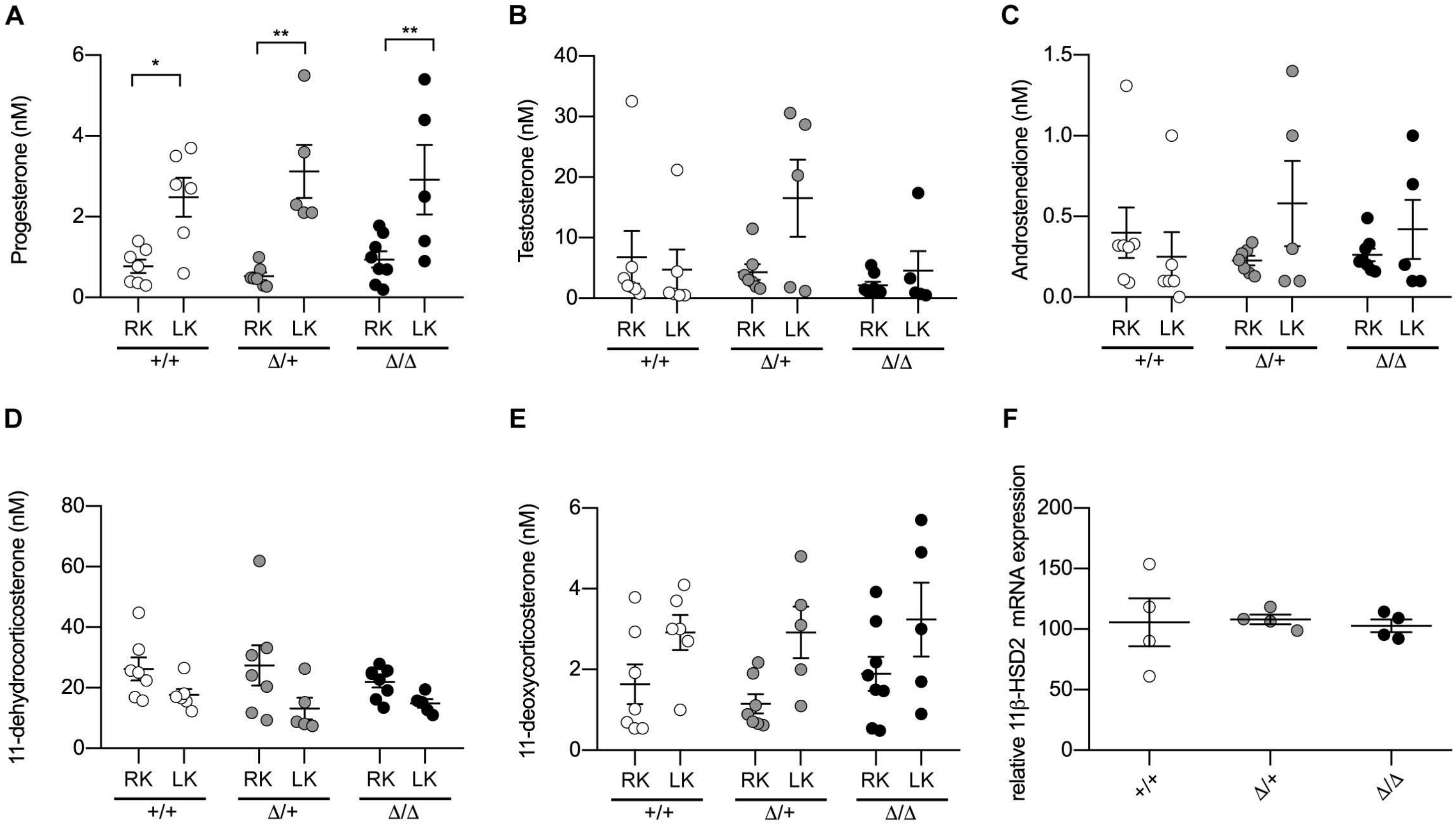

A
B
C
D
E
F
Progesterone (nM)
Testosterone (nM)
Androstenedione (nM)
11-dehydrocorticosterone (nM)
11-deoxycorticosterone (nM)
relative 11β-HSD2 mRNA expression
*
**
**
RK
LK
+/+
Δ/+
Δ/Δ

Figure S7

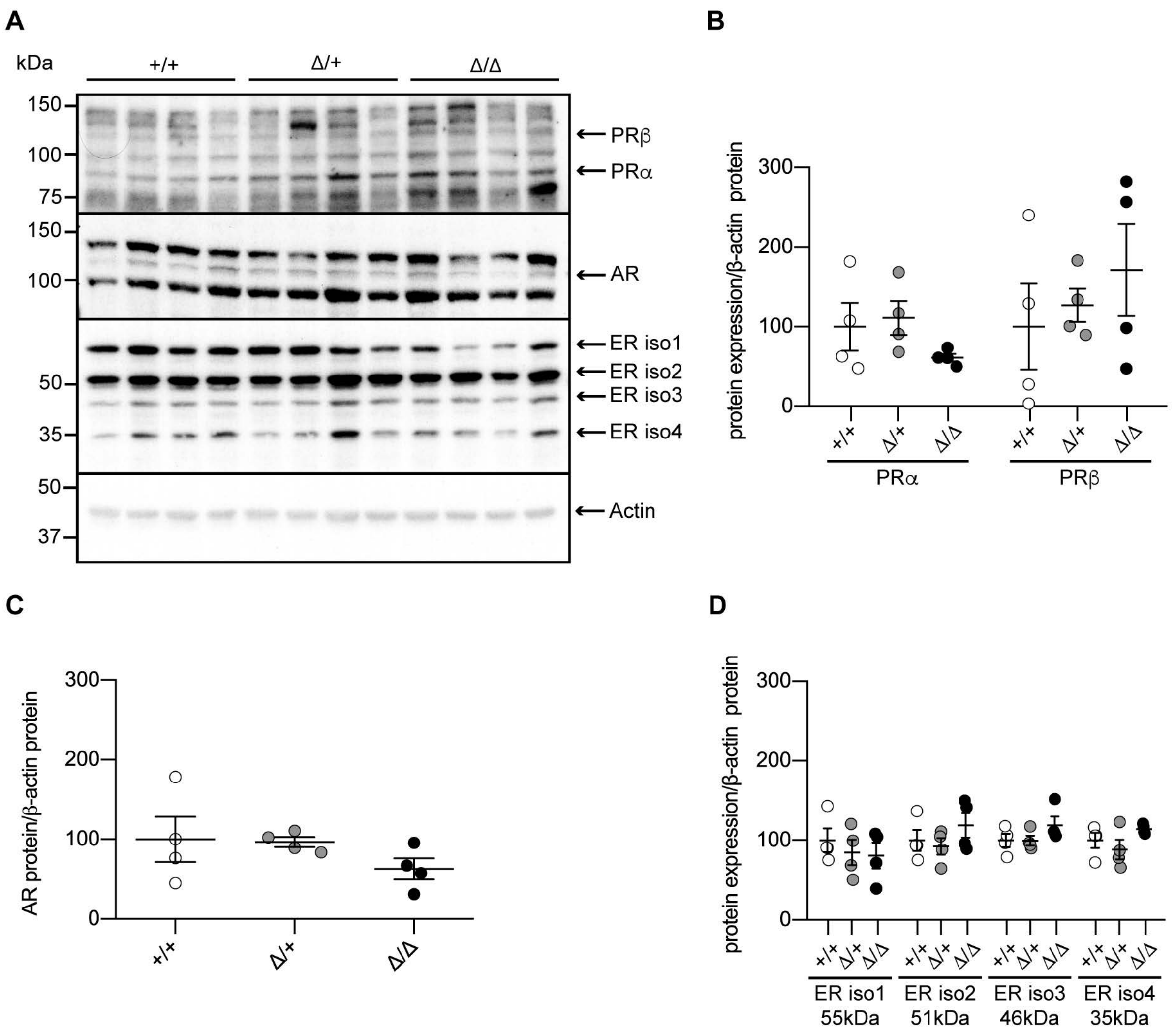
A
kDa
+/+
Δ/+
Δ/Δ
150
100
75
150
100
50
35
50
37
PRβ
PRα
AR
ER iso1
ER iso2
ER iso3
ER iso4
Actin
B
protein expression/β-actin protein
300
200
100
0
+/+
Δ/+
Δ/Δ
PRα
PRβ
C
AR protein/β-actin protein
300
200
100
0
+/+
Δ/+
Δ/Δ
D
protein expression/β-actin protein
300
200
100
0
ER iso1
55kDa
ER iso2
51kDa
ER iso3
46kDa
ER iso4
35kDa

Table S1 : Daily urinary Na^+ excretion (mmol/day) for CAP2/*Tmprss4* mice after 2 days under regular K^+ diet (days 1 and 2) and 4 days (days 3-6) under K^+-deficient diet.

	Day 1	Day 2	Day 3	Day 4	Day 5	Day 6
+/+ 1	0.03	0.01	0.22	0.36	0.54	0.36
+/+ 2	0.01	0.01	0.45	0.60	0.59	0.53
+/+ 3	0.03	0.02	0.20	0.30	0.31	0.20
+/+ 4	0.01	0.01	0.45	0.34	0.26	0.24
+/+ 5	0.01	0.01	0.30	0.38	0.35	0.30
+/+ 6	0.03	0.03	0.28	0.38	0.25	0.18
Δ/+ 1	0.02	0.07	0.30	0.45	0.65	0.61
Δ/+ 2	0.03	0.02	0.35	0.46	0.44	0.50
Δ/+ 3	0.01	0.03	0.25	0.32	0.22	0.19
Δ/+ 4	0.02	0.03	0.06	0.13	0.24	0.22
Δ/+ 5	0.02	0.03	0.08	0.12	0.14	0.18
Δ/+ 6	0.02	0.02	0.09	0.13	0.17	0.18
Δ/Δ 1	0.01	0.03	0.36	0.57	0.44	0.40
Δ/Δ 2	0.01	0.01	0.03	0.21	0.17	0.37
Δ/Δ 3	0.02	0.02	0.10	0.12	0.15	0.13
Δ/Δ 4	0.03	0.02	0.33	0.19	0.19	0.23
Δ/Δ 5	0.03	0.02	0.11	0.14	0.13	0.12
Δ/Δ 6	0.02	0.02	0.17	0.19	0.16	0.22

Table S2 : Daily urinary K^+ excretion (mmol/day) for CAP2/*Tmprss4* mice after 2 days under regular K^+ diet (days 1 and 2) and 4 days (days 3-6) under K^+-deficient diet.

	Day 1	Day 2	Day 3	Day 4	Day 5	Day 6
+/+ 1	0.06	0.05	0.02	0.03	0.03	0.04
+/+ 2	0.02	0.05	0.03	0.02	0.02	0.05
+/+ 3	0.07	0.07	0.01	0.02	0.02	0.03
+/+ 4	0.01	0.02	0.01	0.01	0.00	0.01
+/+ 5	0.03	0.03	0.01	0.01	0.01	0.01
+/+ 6	0.07	0.06	0.03	0.02	0.01	0.02
Δ/+ 1	0.05	0.06	0.03	0.01	0.04	0.05
Δ/+ 2	0.08	0.07	0.02	0.03	0.03	0.05
Δ/+ 3	0.04	0.04	0.01	0.02	0.02	0.03
Δ/+ 4	0.06	0.08	0.03	0.03	0.05	0.03
Δ/+ 5	0.06	0.07	0.02	0.02	0.03	0.05
Δ/+ 6	0.05	0.07	0.02	0.02	0.01	0.03
Δ/Δ 1	0.07	0.06	0.01	0.01	0.02	0.06
Δ/Δ 2	0.03	0.03	0.01	0.02	0.02	0.03
Δ/Δ 3	0.05	0.05	0.02	0.01	0.01	0.03
Δ/Δ 4	0.08	0.07	0.02	0.01	0.03	0.03
Δ/Δ 5	0.07	0.06	0.01	0.01	0.02	0.03
Δ/Δ 6	0.06	0.06	0.02	0.01	0.01	0.04

Table S3 : Daily urinary Na^+ excretion (mmol/day) for $Nr3c1^{Pax8/LC1}$ mice after 2 days under regular K^+ diet (days 1 and 2) and 4 days (days 3-6) under K^+-deficient diet.

	Day 1	Day 2	Day 3	Day 4	Day 5	Day 6
Control 1	0.33	0.22	0.32	0.40	0.35	0.31
Control 2	0.26	0.19	0.19	0.26	0.22	0.30
Control 3	0.11	0.14	0.32	0.34	0.29	0.26
Control 4	0.23	0.07	0.36	0.29	0.24	0.16
Control 5	0.14	0.10	0.35	0.31	0.31	0.24
Control 6	0.15	0.12	0.28	0.34	0.29	0.29
$Nr3c1^{Pax8/LC1}$ 1	0.12	0.13	0.20	0.19	0.23	0.18
$Nr3c1^{Pax8/LC1}$ 2	0.12	0.11	0.21	0.23	0.23	0.20
$Nr3c1^{Pax8/LC1}$ 3	0.10	0.09	0.07	0.20	0.22	0.19
$Nr3c1^{Pax8/LC1}$ 4	0.11	0.10	0.21	0.19	0.21	0.19
$Nr3c1^{Pax8/LC1}$ 5	0.10	0.12	0.13	0.18	0.21	0.12
$Nr3c1^{Pax8/LC1}$ 6	0.05	0.11	0.18	0.18	0.25	0.16

Table S4 : Daily urinary K^+ excretion (mmol/day) for $Nr3c1^{Pax8/LC1}$ mice after 2 days under regular K^+ diet (days 1 and 2) and 4 days (days 3-6) under K^+-deficient diet.

	Day 1	Day 2	Day 3	Day 4	Day 5	Day 6
Control 1	0.10	0.09	0.02	0.01	0.00	0.03
Control 2	0.08	0.09	0.01	0.01	0.01	0.02
Control 3	0.03	0.08	0.02	0.01	0.00	0.02
Control 4	0.03	0.02	0.01	0.01	0.01	0.02
Control 5	0.05	0.06	0.01	0.01	0.01	0.02
Control 6	0.06	0.05	0.01	0.01	0.01	0.02
$Nr3c1^{Pax8/LC1}$ 1	0.05	0.05	0.01	0.01	0.00	0.01
$Nr3c1^{Pax8/LC1}$ 2	0.04	0.06	0.00	0.00	0.00	0.01
$Nr3c1^{Pax8/LC1}$ 3	0.03	0.05	0.00	0.00	0.01	0.01
$Nr3c1^{Pax8/LC1}$ 4	0.02	0.04	0.01	0.00	0.00	0.01
$Nr3c1^{Pax8/LC1}$ 5	0.03	0.05	0.00	0.00	0.00	0.01
$Nr3c1^{Pax8/LC1}$ 6	0.07	0.04	0.00	0.00	0.00	0.02

Uncropped Western blots part 1

Fig. 3C

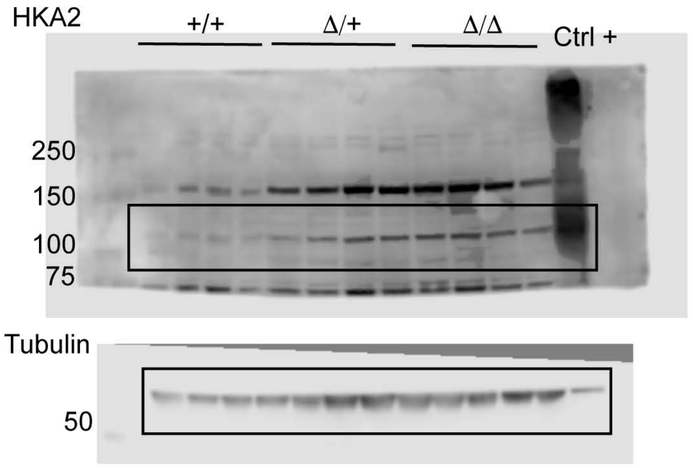

Fig. 4A

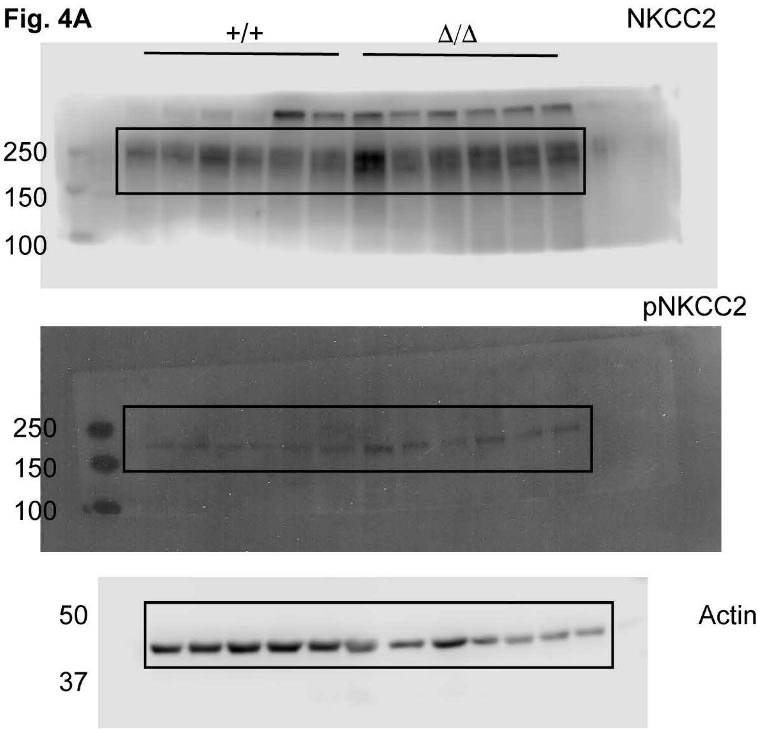

Fig. 4C

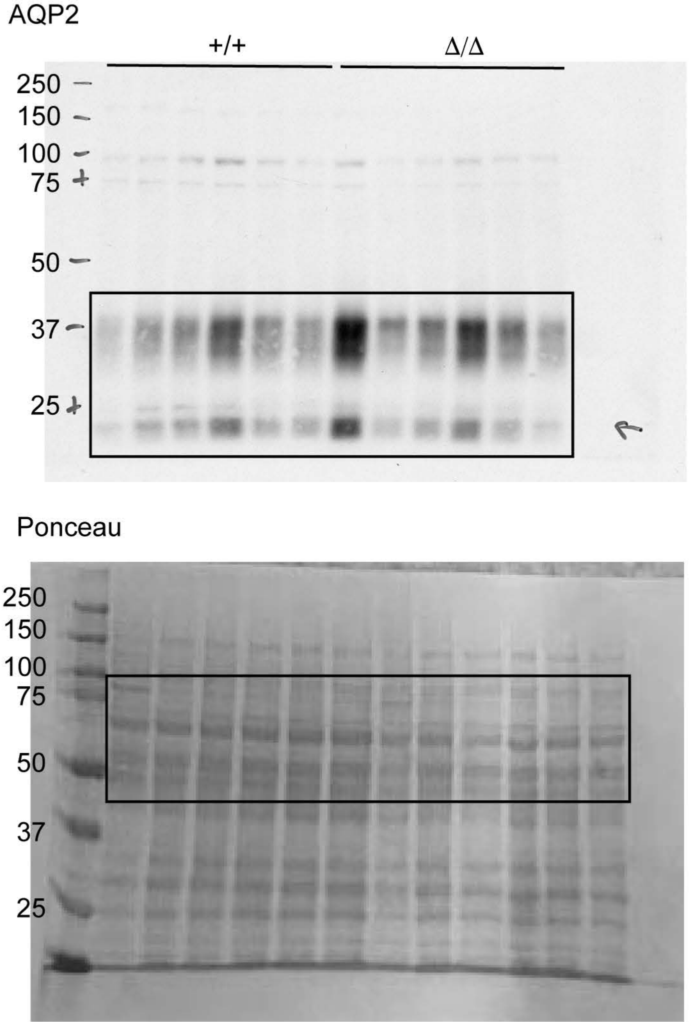

Fig. 5C

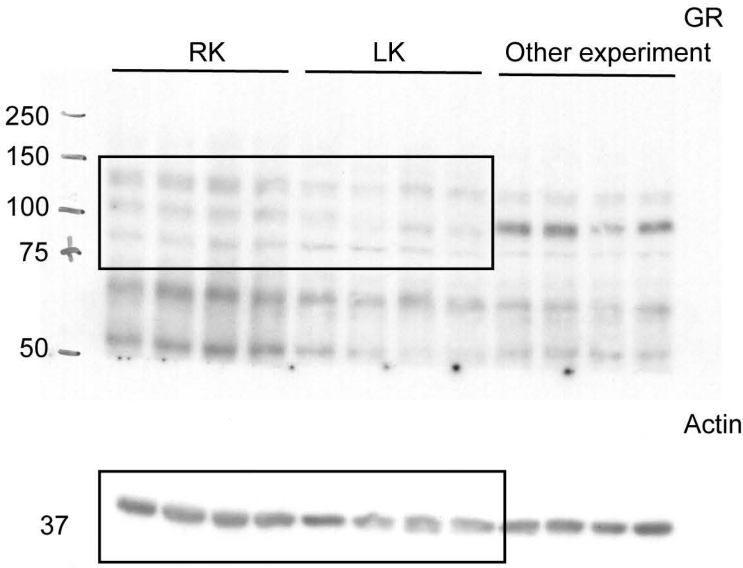

Uncropped Western blots part 2

Fig. 5E

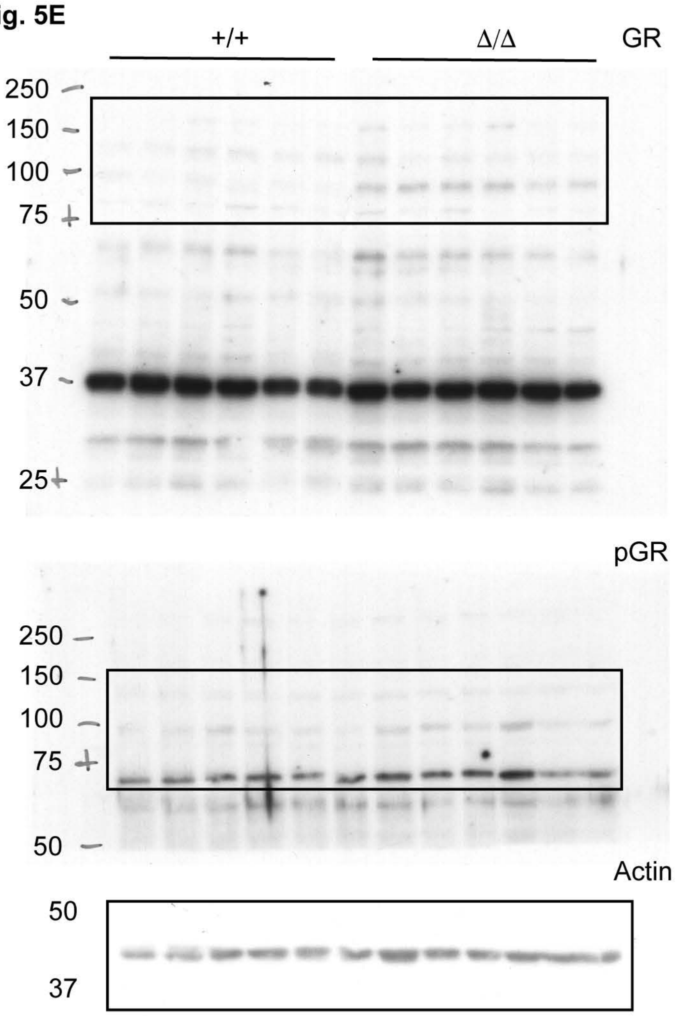

Fig. 5G

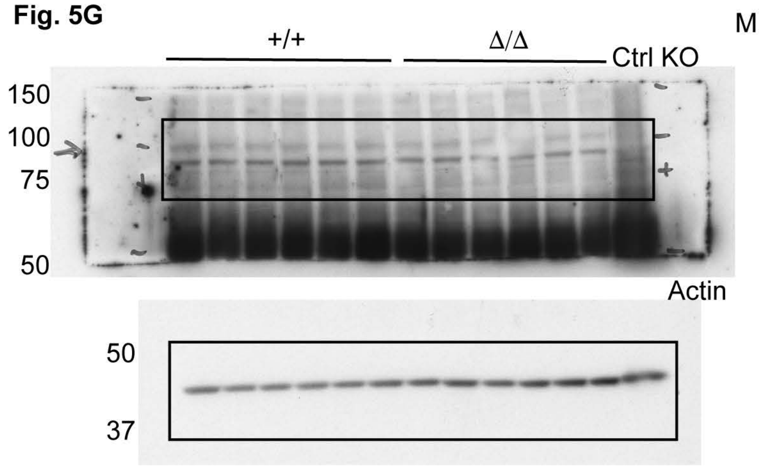

Fig. 7B

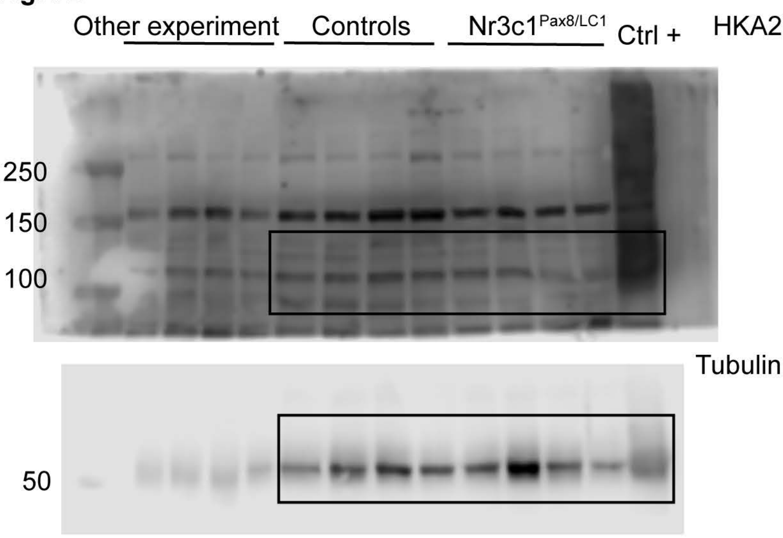

Fig. 7D

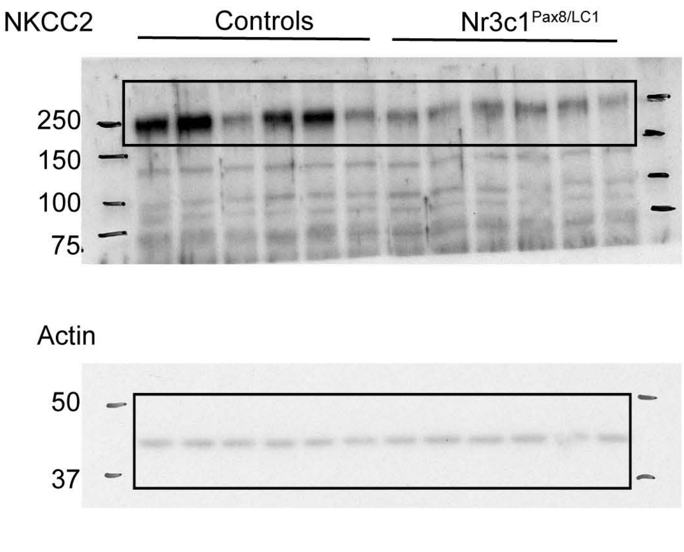

Fig. 7F

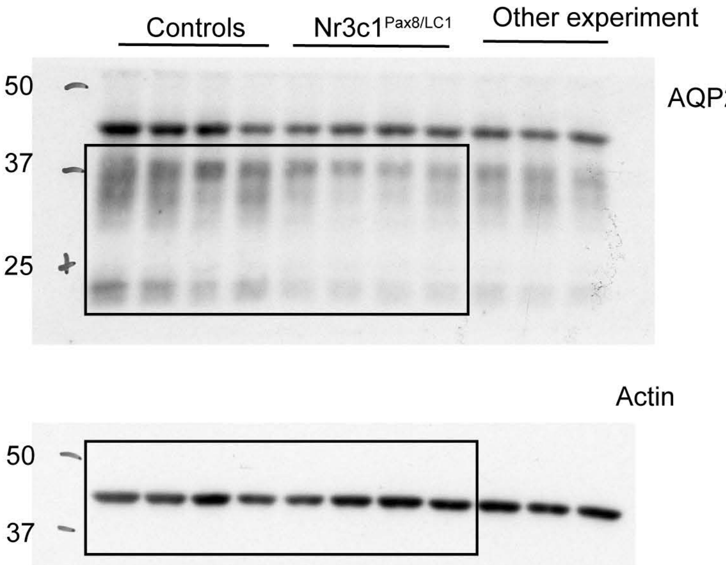

Uncropped Western blots part 3

Fig. S4D

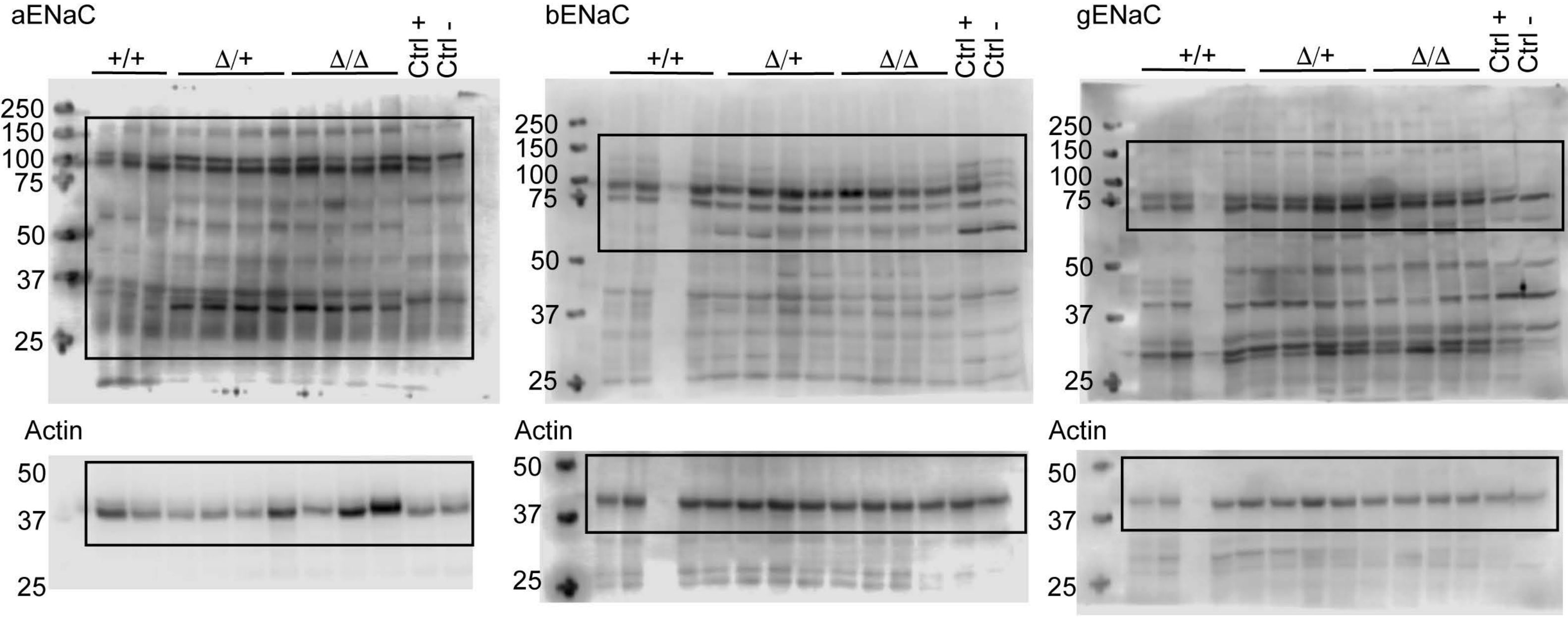

Fig. S4F

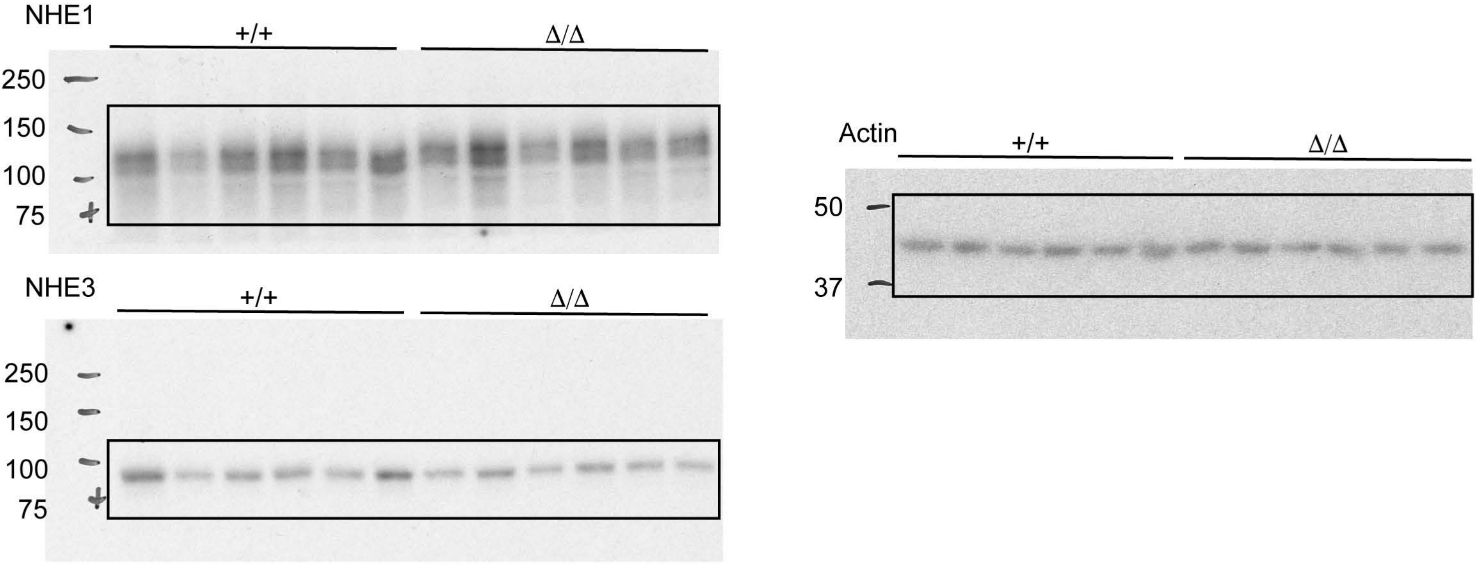

Fig. S5A

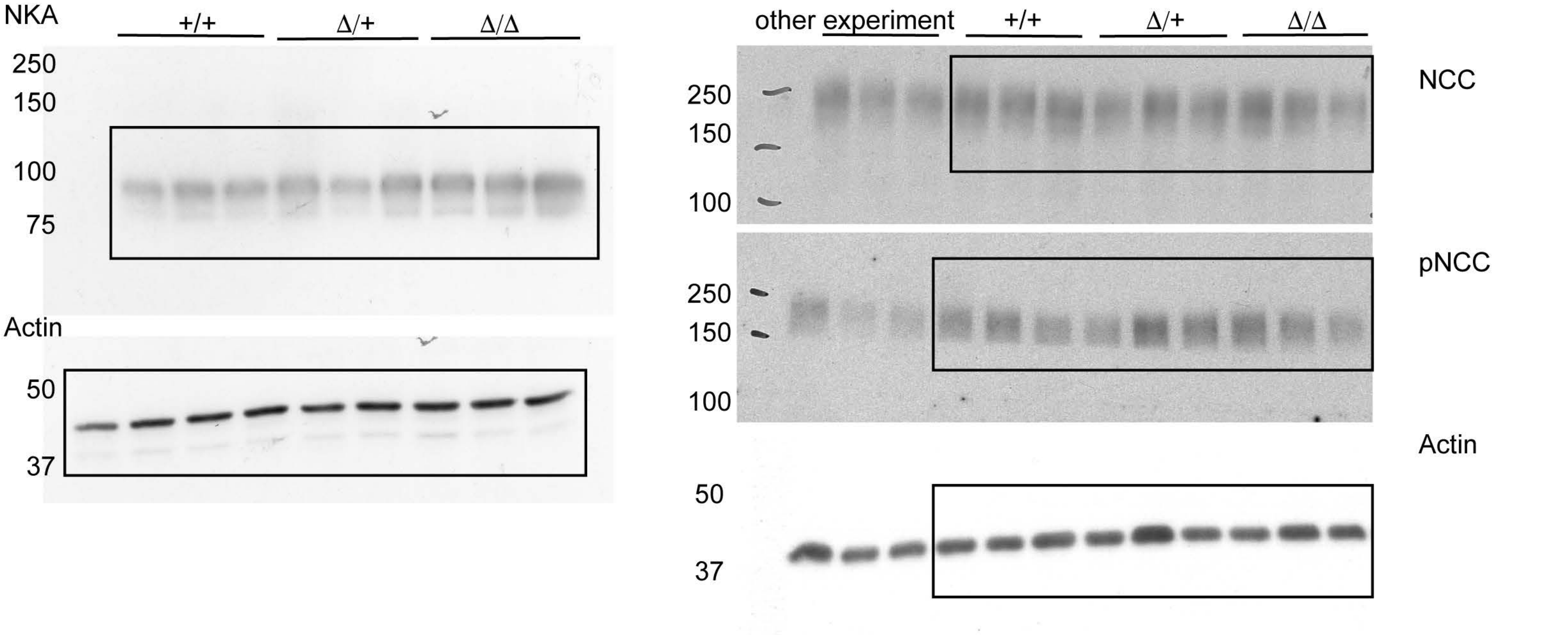

Uncropped Western blots part 4

Fig. S7A

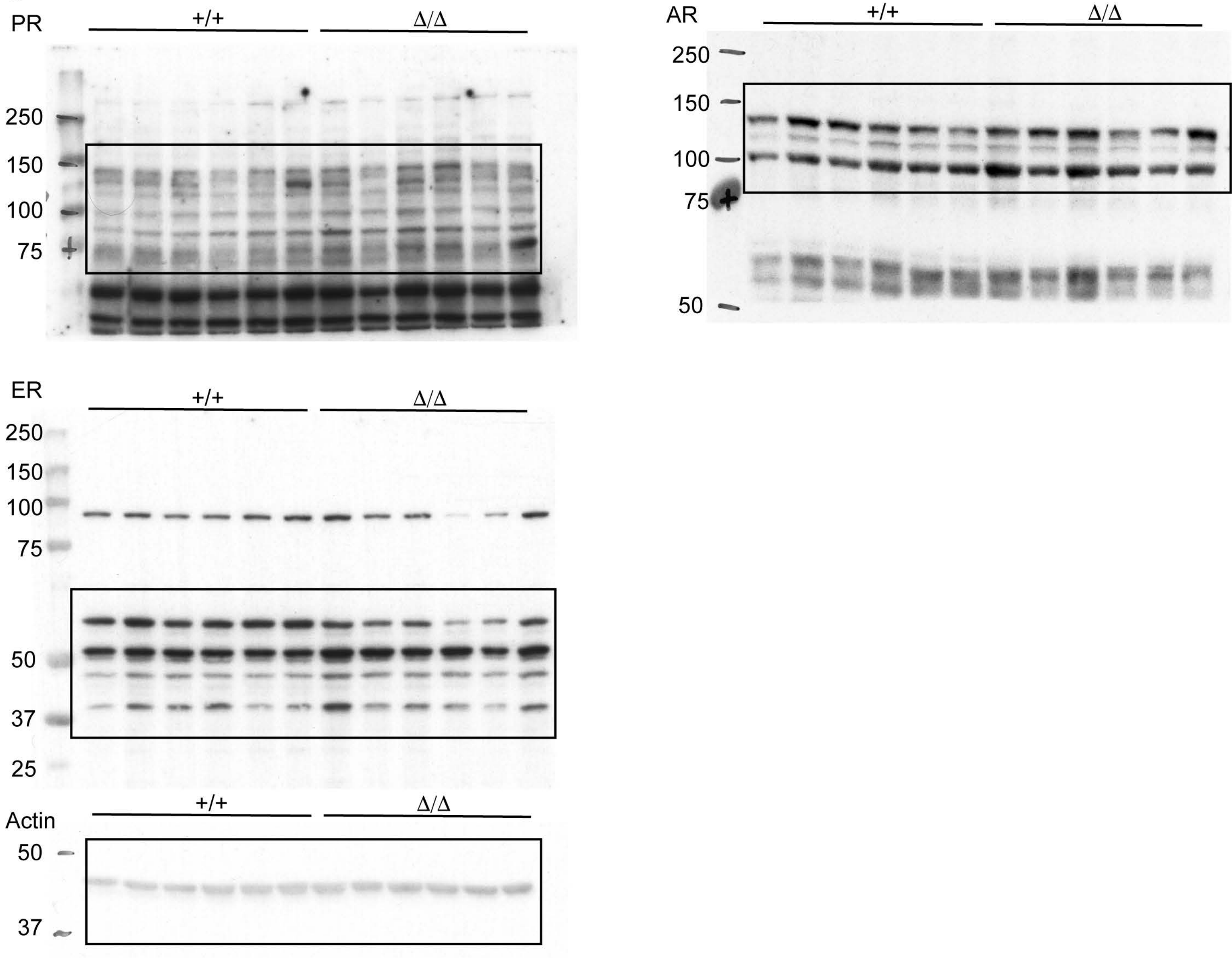

Supplement: Supplementary file 1 — Supplementary information [file 41598_2019_55995_MOESM1_ESM.pdf]
